# Structural Diversity and Analytical Characterization of Acylhomocarnitines

**DOI:** 10.1021/acs.jproteome.5c01255

**Published:** 2026-05-13

**Authors:** Jaclyn Weinberg, William J. Crandall, Zachery Ryan Jarrell, Gahyun Lim, Ken Liu, Ho Young Lee, Shakshi Patel, Camilo Anthony Gacasan, Young-Mi Go, Dean P. Jones

**Affiliations:** † Division of Pulmonary, Allergy, Critical Care and Sleep Medicine, Department of Medicine, 12239 Emory University School of Medicine, Atlanta, Georgia 30322, United States; ‡ Department of Chemistry, 1371 Emory University, Atlanta, Georgia 30322, United States; § Department of Biochemistry, Department of Medicine, Emory University, Atlanta, Georgia 30322, United States; ∥ Division of Gastroenterology, Hepatology, and Nutrition, Department of Medicine, Emory University, Atlanta, Georgia 30322, United States

**Keywords:** acylcarnitine, carnitine, chromatography, energy-resolved, fatty acid metabolism, HILIC, homocarnitine, isomer, mass spectrometry, valerobetaine

## Abstract

Homocarnitine is a five-carbon analog of carnitine produced in mammals through hydroxylation of the microbiome-derived metabolite δ-valerobetaine. Here, we describe liquid chromatography–mass spectrometry methods for the measurement of fatty acyl-homocarnitines, a previously uncharacterized family of mammalian metabolites. These acyl-homocarnitines are homologs of acyl-carnitines, in which the fatty acid is extended by one carbon. We show that short-chain fatty acyl-CoAs are converted to corresponding acyl-homocarnitines by carnitine acetyltransferase and that these enzyme-generated standards exhibit retention times and ion dissociation patterns identical to acyl-homocarnitines produced by mammalian cells. *In vitro* ^13^C_3_-homocarnitine isotope tracer studies showed that mammalian cells produce short-, medium-, and long-chain acyl-homocarnitines. Ion dissociation analyses established diagnostic product ions to distinguish acyl-homocarnitines from isomeric acyl-carnitines. Sample preparation and chromatographic methods are provided to separate and analyze isomers in extracts of mouse tissues. These findings expand knowledge of carnitine analogs and establish analytical strategies to differentiate acyl-homocarnitines from isomeric acyl-carnitines.

## Graphical abstract

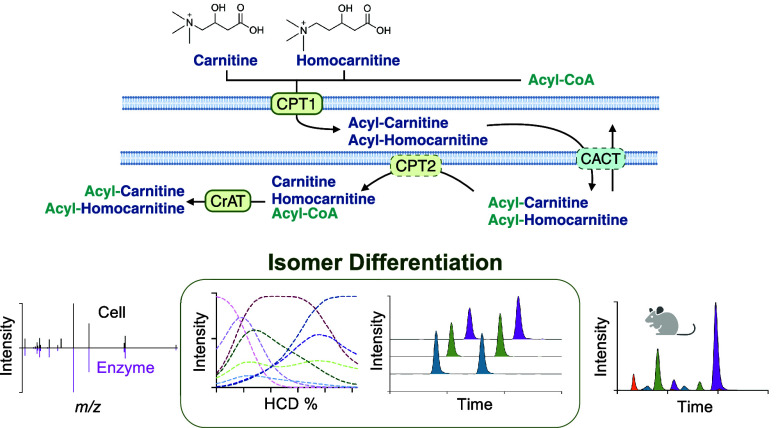

## Introduction

Homocarnitine is a five-carbon structural analog of carnitine, an essential carrier for fatty acids in mitochondrial β-oxidation. Homocarnitine is derived from mammalian hydroxylation of the microbiome metabolite, δ-valerobetaine, and thus represents a metabolic bridge between the intestinal microbiome and mammalian host tissues.^1^ In humans, δ-valerobetaine has been associated with disruptions to fatty acid oxidation and metabolic diseases, including obesity,^2^ diabetes,^3,4^ cardiovascular disease,^5^ and nonalcoholic fatty liver,^2,6^ through disruption of carnitine homeostasis.^1,2,6^ As an analog of carnitine, homocarnitine undergoes acylation by carnitine acyltransferases to form an acetyl- and palmitoyl-homocarnitine.^1^ Through pharmacological inhibition and isotope tracing, we previously demonstrated that carnitine palmitoyltransferase 1A (CPT1A) catalyzes the formation of palmitoyl-homocarnitine (C16) *in vitro*, and we subsequently detected this metabolite in mouse tissues. We also detected acetyl-homocarnitine (C2) *in vitro*, in mouse samples, and in human plasma.^1^

Our initial studies revealed challenges in separating even-chain acyl-homocarnitines from more abundant and isomeric odd-chain acyl-carnitines, supporting the need for improved analytical strategies to differentiate acyl-homocarnitines from acyl-carnitines.^1^ For example, acetyl-homocarnitine (C2) shares the same monoisotopic mass as propionylcarnitine (C3). This structural similarity can result in coelution on common hydrophilic and reversed-phase chromatography platforms and necessitates careful interpretation of MS^2^ ion dissociation patterns for confident identification. Furthermore, no standards are commercially available to validate acyl-homocarnitine ion dissociation patterns or retention times. Combining our previous observations from an *in vitro* fatty acid isotope tracing study and endogenous signals in cells, mice, and humans, we found that fragments at *m*/*z* 99 and 158 were likely unique to acyl-homocarnitines, whereas structurally analogous fragments at *m*/*z* 85 and 144 were specific to acyl-carnitines. Conversely, we found that reliance on product ions at *m*/*z* 60 and 159 to annotate acyl-carnitines could result in acyl-homocarnitines being overlooked.^1^

On the basis of our evidence for acetyl-homocarnitine and palmitoyl-homocarnitine, the goals of the present study were to define the structural diversity of the acyl-homocarnitine family and establish chromatographic and mass spectrometric methods to differentiate acyl-homocarnitines from isomeric acyl-carnitines. Using enzyme assays, we demonstrate acylation of homocarnitine by carnitine acetyltransferase (CrAT) and generate standards for short-chain acyl homocarnitines. Detailed analyses of *in vitro* ^13^C_3_-homocarnitine isotope tracer studies show dose-dependent formation of short-, medium-, and long-chain acyl homocarnitines in mammalian cells. Diagnostic product ions and optimal collision energies for isomer differentiation are presented, and optimized liquid chromatography (LC) conditions show baseline separation of isomers. Finally, we present a sample preparation workflow for the application of an LC-MS^2^ method to acyl-homocarnitine measurement in mouse tissue.

## Materials and Methods

### Materials

All solvents and chemical additives used for metabolite extraction and liquid chromatography–mass spectrometry (LCMS) analysis were LCMS-grade. The following were purchased from Sigma-Aldrich: l-Carnitine (C0158), γ-butyrobetaine (403245), acetyl-coenzyme A (A2056), propionyl-coenzyme A (P5397), ammonium formate (70221), ammonium acetate (73594), Dulbecco’s modified Eagle medium (DMEM) (D5796), Gibco Antibiotic-Antimycotic 100X (15240062), Gibco Horse Serum (16050130), and Gibco Insulin-Transferrin-Selenium (ITS-G) (41400045). δ-Valerobetaine (DC26004) was purchased from DC Chemicals (Shanghai, China). Valeryl-l-carnitine (26563) and butyryl-coenzyme A (27865) were purchased from Cayman Chemicals (Ann Arbor, Michigan, USA). Stable *d*_9_ or *d*_3_ isotopically labeled acylcarnitine standards were purchased from Cambridge Isotope Laboratories, including C0, C2, C3, C4, C5 (isovaleryl), C8, C14, and C16. Homocarnitine and ^13^C_3_-homocarnitine were purchased as a custom synthesis project from Chiroblock GmbH (Bitterfeld-Wolfen, Germany). *d*_9_-δ-Valerobetaine (GC72846) was purchased from GLPBIO (Montclair, CA, USA). *d*_9_-γ-Butyrobetaine was purchased from LGC (TRC-B759497). Waters Oasis MCX 6 cc Vac 150 mg sorbent (60 μm) solid-phase extraction cartridges (41115712) were purchased from Waters (Milford, MA, USA). Human AC16 (CRL-3578) cells and ATCC F-12K medium (30–2004) were purchased from ATCC. BenchMark Fetal bovine serum (100–106) was purchased from GeminiBio (Sacramento, CA, USA). Western Diet (D12079B) for mice was purchased from Research Diets (Brunswick, NJ, USA).

### Experimental Animals

Mouse heart samples were obtained from an ongoing study on metabolic health in aging using C57BL/6J mice sourced from Charles River and provided by the National Institute on Aging. The study was conducted following Emory University’s Institutional Animal Care and Use Committee (IACUC) protocol (PROTO202300105), and experiments aligned with relevant guidelines and regulations. Female mice at 18–20 months old were fed a Western diet (40% kcal from fat) and given *ad libitum* access to drinking water with or without 1.62 mM δ-valerobetaine for 12 weeks. Mice were monitored throughout for signs of illness or excessive weight loss by IACUC-defined humane end points. At the end of the study, mice were euthanized via CO_2_ asphyxiation followed by cervical dislocation. Heart tissue was collected and stored at −80 °C until further processing.

### Huh7 Cell Line

Huh7 cells were provided by Dr. Arash Grakoui of Emory University and verified by the Emory Integrated Genomics Core facility.^7^ Human hepatoma Huh7 cells were plated on 6-well plates with DMEM containing 10% FBS and 1% penicillin/streptomycin. To test for cell-generation of C2, C3, and C4-homocarnitine, cells were treated with media containing 0 or 50 μM homocarnitine at 80% confluency and incubated for 18 h (*n* = 6 each). For the isotope tracing studies, cells were treated with media containing ^13^C_3_-homocarnitine at 0, 0.1, 0.5, 1, 3, 10, and 30 μM. Cells were treated with 0, 0.5, 1.5, 5, 15, 50, and 100 μM ^13^C_3_-homocarnitine for follow-up studies. After an 18 h incubation, the cells were washed twice with ice-cold HBSS and extracted by scraping with 200 μL ice-cold HPLC-grade acetonitrile:water (80:20) on ice. Extracts were transferred to microcentrifuge tubes for centrifugation at 20,817*g* for 10 min at 4 °C. Supernatants were transferred to autosampler vials and stored at 4 °C for LCMS analysis. Samples were injected 3 times each.

### AC16 Cell Line

Human AC16 (CRL-3578) cells, derived from primary adult ventricular tissue, were cultured for four passages in 50% DMEM/50% F-12K media with 10% FBS and 1% Antibiotic-Antimycotic. Cells were then differentiated into mature cardiomyocytes with an expected increase in dependence on fatty acid oxidation compared to glucose oxidation.^8^ Differentiation was induced by switching the media to 50% DMEM/50% F-12K with 2% horse serum, 1x insulin-transferrin-selenium, and 1% antibiotic-antimycotic. After 1 week, differentiation was observed with elongation of cells and organization of rod-shaped cells into a parallel pattern.^9^ At 80% confluency, cells were incubated in 6-well plates with ^13^C_3_-homocarnitine at 0, 0.5, 1.5, 5, 15, 50, and 100 μM for 18 h (*n* = 6). After treatment, cells were prepared for LCMS as described for the Huh7 cells.

### Carnitine Acetyltransferase (CrAT) Production

Murine CrAT pET28a plasmid was a gift from Dr. Liang Tong (Columbia University, NY), and sequence confirmation was performed. The plasmid was transformed into BL21 DE3 *Escherichia coli* with terrific broth at 37 °C with shaking. When the concentration reached OD_600_, the temperature was reduced to 18 °C and 0.5 isopropyl-1-thio-D-galactopyranoside was added for an overnight incubation. The cells then underwent centrifugation at 3500*g* for 20 min. Cell pellets were stored at −80 °C. For purification, the cells were lysed with 150 mM NaCl, Tris-HCl pH 7.4, 5% glycerol, 25 mM imidazole, 5 mM β-mercaptoethanol, 100 μM PMSF, DNAase A, and lysosome, and underwent centrifugation at 18,000*g* for 1 h. The supernatant was transferred to an immobilized metal affinity chromatography column. Buffer A consisted of 150 mM NaCl, 20 mM Tris, 20 mM imidazole, and 5% glycerol at pH 7.5. Buffer B consisted of 150 mM NaCl, 20 mM Tris, 250 mM imidazole, and 5% glycerol at pH 7.5. CrAT-containing fractions were subjected to a Superdex 75pg 16/600 mm size exclusion column (28989333, Cytiva HiLoad) with 20 mM HEPES, pH 8.2, 200 mM NaCl, 0.05% Tween-20, and 0.5 mM TCEP. The resulting CrAT protein was aliquoted and frozen at −80 °C until experiments.

### Generation of Acyl-Carnitines and Acyl-Homocarnitines with CrAT

The reaction was carried out in Tris-HCl buffer at pH 7.8. The reaction mixture contained 250 μM carnitine or homocarnitine with 100 μM acetyl-, propionyl-, or butyryl-CoA. The reaction was initiated with vehicle or 50 μg/mL CrAT in buffer and incubated at 37 °C for 30 min.^10^ The reaction was terminated with ice-cold methanol at 7x reaction volume. The extracts underwent centrifugation at 20,817*g* for 10 min at 4 °C. Supernatants were transferred to autosampler vials for LCMS analysis.

### Standards

A stock solution of internal standards was created for tissue extractions by mixing equal volumes of *d*_3_-valerobetaine, *d*_3_-butyrobetaine, *d*_3_-carnitine, *d*_3_-acetylcarnitine, *d*_3_-propionylcarnitine, *d*_3_-butyrylcarnitine, *d*_9_-isovalerylcarnitine, *d*_3_-octanoylcarnitine, *d*_9_-myristoylcarnitine, and *d*_3_-palmitoylcarnitine. All compounds were purchased preweighed at 5 mg. Each standard was dissolved in 1.8 mL of 90% methanol with 0.1% formic acid and further diluted 30x. The internal standard solution was further diluted 200x into 2:1 acetonitrile:water at 46.3 ng/mL for tissue extractions.

### Liquid Chromatography

A Thermo Scientific Vanquish Duo UHPLC was used for HILIC and C18 methods. The autosampler was chilled to 4 °C, and the column compartment was heated to 45 °C. The needle was washed before and after every injection for 30 s with 50% methanol with 0.1% formic acid. A double blank solution of 90% methanol with 0.1% formic acid was injected after samples to test for carryover. Blanks with internal standard were used for retention time, signal intensity, and mass accuracy quality control.

The general HILIC method consisted of an Acquity BEH Amide HILIC column, 2.1 mm × 100 mm, 1.7 μm (Waters, 186004801) coupled to a ACQUITY UPLC BEH Amide VanGuard Pre-Column, 2.1 mm × 5 mm, 1.8 μm (Waters, 186004799). Buffer A was water with 1 mM ammonium acetate and 0.1% formic acid, and Buffer B was 95% acetonitrile with 1 mM ammonium acetate and 0.1% formic acid. The flow rate was 0.25 mL/min. The gradient started at 90% for 1.5 min, ramped to 20% B over 6 min, and held for 2.5 min.

The C18 chromatography consisted of a Hypersil GOLD C18 column, 2.1 mm × 100 mm, 1.9 μm (Thermo Scientific, 25003–102130) coupled to an Acquity UPLC CSH TS3 C18 VanGuard Pre-Column, 2.1 mm × 5 mm, 1.9 μm (Waters, 186003976). Buffer A was 0.1% formic acid in water, and Buffer B was 99% acetonitrile with 0.1% formic acid. The flow rate was 0.35 mL/min, and the gradient started at 4% B for 0.75 min and ramped to 99% over 6 min, and then held for 3.25 min.

### HILIC Chromatography for Isomer Separation

For separation of the short-chain acyl-homocarnitine and acyl-carnitine isomers and measurement of related betaines, an optimized HILIC method consisted of an Accucore HILIC column, 2.1 × 50 mm, 2.6 μm (Thermo Scientific, 17526-052130) with an Accucore HILIC guard, 2.1 × 10 mm, 2.6 μm (Thermo Scientific, 17526-012105). The flow rate was 0.3 mL/min. The column compartment was 45 °C. Buffer A was 25 mM ammonium formate in water, and Buffer B was 95% acetonitrile and 5% water. The gradient started at 90% B for 0.6 min, ramped to 40% B from 0.6 to 7.55 min, held from 7.55 to 8.15 min, then ramped to 30% B from 8.15 to 9.15 min, and held for 1 min. Before the next run, buffer B was ramped back to 90% over 1 min and held for 4 min.

### Mass Spectrometry

The UHPLC was connected to a Thermo Scientific ID-X Orbitrap mass spectrometer for most experiments. The AC16 cell experiment was run on a Thermo Scientific Orbitrap Exploris 120. Positive electrospray ionization was set to 3.5 kV. Sheath gas was set to 50 (in arbitrary units), auxiliary gas at 10, and sweep gas at 1. The vaporizer temperature was set at 300 °C, and the ion transfer tube temperature was set at 350 °C. MS^1^ scans were collected with the Orbitrap detector at 120k or 60k resolution. MS^2^ or MS^3^ scans were acquired at 15k resolution. ACG targeted and max injection time were set to auto. Total cycle time was set to 0.6 s. A targeted inclusion list was used with an isolation window of 15 ppm. Ion dissociation was set to normalized HCD at 35% for targeted ion dissociation. For the energy-resolved mass spectrometry (ERMS) experiments, HCD or CID collision energy % was ramped from 10 to 62 with steps of 2 increments. For CID methods, the CID activation time was 10 ms and Activation Q was 0.25. Each ERMS method contained three collision energies (e.g., 10, 12, 14%), and the total cycle time was set to 9 scans. For HCD, the normalized energies on the ID-X can be converted to absolute energies (eV) with this equation: (normalized collision energy × isolation center)/(500 *m*/*z* × charge factor). The isolation center is the target *m*/*z*. The charge factor for our singly charged targets is 1.

### Liquid Extraction of Tissue

Mouse heart was cut to ∼30 mg. An extraction solution of 2:1 acetonitrile:water with internal standard solution was prepared. The tissue was treated with the extraction solution at 12 μL/mg tissue, vortexed, and thawed for 10 min. Next, the samples were placed on dry ice to freeze for 10 min. A total of 3 thawing, freezing, and vortexing cycles were performed. Next, samples underwent centrifugation at 25,830*g* for 10 min at 4 °C. Supernatants were dried at 45 °C in a vacuum centrifuge. The pellets were reconstituted in 4 mL 2% formic acid in methanol, vortexed, sonicated for 2 min, and then underwent centrifugation for 5 min at 25,830*g*. Supernatants then underwent solid-phase extraction.

### Solid-Phase Extraction of Samples

The solid-phase extraction routine was adapted from Waters protocols, and He et al.^11^ The Waters MCX cartridges were preconditioned with 3 mL methanol and equilibrated with 3 mL 2% formic acid in water. The metabolite extract was loaded slowly and then washed with 3 mL 2% formic acid, 3 mL methanol, and eluted with 3 mL 5% ammonium hydroxide in methanol. The supernatant was collected and dried at 45 °C. The samples were reconstituted in 120 μL methanol, vortexed, sonicated for 2 min, and underwent centrifugation for 5 min at 25,830*g*. The supernatant was transferred to autosampler vials for LCMS.

### Software and Analysis

xCalibur (v.4.5.455.18, Thermo Scientific) QualBrowser was used to evaluate MS^2^ and MS^3^ spectra and chromatograms. The MS^n^ spectra shown reflect the average of all scans across a peak. Skyline (v.23.1.0.0455, MacCoss Lab)^12^ was used for peak area calculation. Peak areas from technical triplicates were median-summarized. Predicted values for the polar surface area (Cactvs v2.3.8.18) and logP (XLogP3 3.0) were obtained from PubChem (release 2025.04.14) for C2-homocarnitine (CID 172913711) and C3-carnitine (CID 107738). Predicted values for logP (CLogP) were also obtained from ChemDraw (v.22.2.0.3348). GraphPad Prism (v.10.6.1) was used for nonlinear regressions and CE_50_ calculations. Biorender.com was used to create the graphical abstract and Figure 5.

## Results and Discussion

### Enzymatic Generation of Acyl-Homocarnitine Standards

Carnitine acetyltransferase (CrAT) was used to generate short-chain acyl-homocarnitines by incubation of homocarnitine with acetyl (C2), propionyl (C3), or butyryl (C4) coenzyme A with or without recombinant mouse CrAT for 30 min. Respective signals for acyl-homocarnitines were detected for C2-homocarnitine (*m*/*z* 218.1387, Δ 0 ppm), C3-homocarnitine (*m*/*z* 232.1551, Δ 3.0 ppm), and C4-homocarnitine (*m*/*z* 246.1700, Δ 0.8 ppm), only with enzyme (Figure 1A; Supporting Information, Figure S1). This confirmed the catalytic activity of CrAT with homocarnitine and verified that CrAT can be used to generate acyl-homocarnitine standards. Acyl-homocarnitines were detected with HILIC in order of decreasing acyl chain length (Figure 1A), similar to the elution pattern of CrAT-generated carnitine analogs, including C4-carnitine (*m*/*z* 232.1543, Δ 0.4 ppm), C3-carnitine (*m*/*z* 218.1387, Δ 0 ppm), and C2-carnitine (*m*/*z* 204.1230, Δ 0 ppm) (Supporting Information, Figure S2).

**Figure 1:**
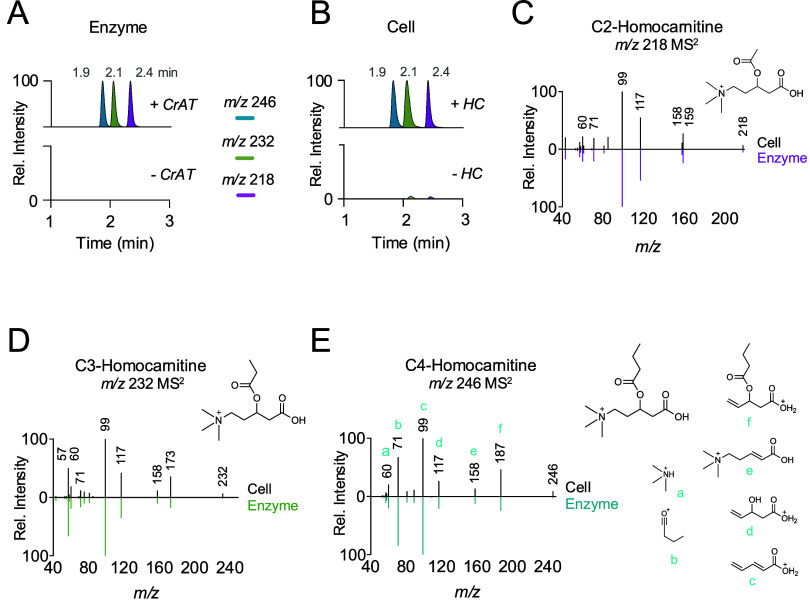
Acyl-homocarnitine retention times and mass spectral signals from enzymatically generated standards. (A) Extracted ion chromatograms from a HILIC/ESI+ method showing enzyme generation of C2-, C3-, and C4-homocarnitine in the presence (top) or absence (bottom) of carnitine acetyltransferase (CrAT). Each peak in the bottom chromatogram is normalized to the same peak in the top chromatogram. *m*/*z* 246.1700 (blue) = butyryl-homocarnitine (C4). *m*/*z* 232.1551 (green) = propionyl-homocarnitine (C3). *m*/*z* 218.1387 (purple) = acetyl-homocarnitine (C2). Replicates and a positive control experiment with carnitine are available (Supporting Information, Figures S1 and S2). (B) Extracted ion chromatograms of *m*/*z* 218 (purple), *m*/*z* 232 (green), and *m*/*z* 246 (blue) from analysis of Huh7 cells treated with 50 μM homocarnitine (top) or vehicle control (bottom) for 18 h. Without homocarnitine, these peaks were predominantly C3-, C4-, C5-carnitine (bottom). With homocarnitine treatment, the peaks were mostly C2-, C3-, and C4 -homocarnitine (top) (see MS^2^ spectra in Supporting Information, Figure S3). Each peak in the bottom chromatogram is normalized to the same peak in the top chromatogram. (C) Mirror plot showing MS^2^ ion dissociation spectra (HCD 35%) of C2-homocarnitine from cells (top; black) and CrAT-generated C2-homocarnitine biological standard from Huh7 cells (bottom; purple). (D) Mirror plot showing MS^2^ spectra (HCD 35%) of C3-homocarnitine from cells (top; black) and CrAT-generated C3-homocarnitine (bottom; green). (E) Mirror plot showing MS^2^ spectra (HCD 35%) of C4-homocarnitine from cells (top; black) and CrAT-generated C4-homocarnitine (bottom; blue) along with proposed product ion structures. Proposed ion dissociation sites are indicated by blue lines, with elbow indicating directionality of resulting fragment. *m*/*z* 57 = 57.0335. *m*/*z* 60 = 60.0809. *m*/*z* 71 = 71.0490. *m*/*z* 99 = 99.0441. *m*/*z* 117 = 117.0547. *m*/*z* 158 = 158.1179. *m*/*z* 159 = 159.0651. Rel. Intensity = relative intensity.

To substantiate signals previously assigned to C2-homocarnitine in cell, mouse, and human samples,^1^ we compared the retention times and MS^2^ spectra of the CrAT-generated acyl-homocarnitine standards to cell-generated acyl-homocarnitines (Figure 1B–E). Huh7 human hepatoma cells incubated with a relatively high concentration of homocarnitine (50 μM) for 18 h (*n* = 6 each) showed expected increases in signals corresponding to C2-homocarnitine (*m*/*z* 218.1387), C3-homocarnitine (232.1551), and C4-homocarnitine (246.1700) compared to the vehicle control (Figure 1B). MS^2^ spectra showed *m*/*z* 60.0809 (C_3_H_10_N^+^), 71.0490 (C_4_H_7_O^+^), 99.0440 (C_5_H_7_O_2_^+^), 117.0547 (C_5_H_9_O_3_^+^), and 158.1178 (C_8_H_16_NO_2_^+^)^1^ consistent with acyl-homocarnitine ion dissociation. Comparison of product ion *m*/*z* and intensity distributions to the spectra from the CrAT-generated standards (Figure 1C–E) substantiated the identification of C2-, C3-, and C4-homocarnitine in cells as well as that of C2-homocarnitine in our previous study.^1^ For comparison, the Huh7 cell vehicle control condition showed predominant signals for endogenous C3-carnitine (*m*/*z* 218.1388; median peak area = 3.2 × 10^6^), C4-carnitine (*m*/*z* 232.1544; median peak area = 3.9 × 10^6^), and C5-carnitine (*m*/*z* 246.1700; median peak area = 2.4 × 10^5^) (Figure 1B). MS^2^ ion dissociation (HCD 35%) produced characteristic spectra for acyl-carnitines with the major product ion at *m*/*z* 85.0284 (C_4_H_5_O_2_^+^) and minor product ions at *m*/*z* 60.0809 (C_3_H_10_N^+^) and 144.1016 (C_7_H_14_NO_2_^+^)^13–16^ (Supporting Information, Figure S3). Comparison of MS^2^ spectra and retention times of these cell extract acyl-carnitines to the CrAT-generated acyl-carnitine signals further verified these identifications. It should be noted that due to coelution, some acyl-carnitine product ions (e.g., *m*/*z* 85) were present even with homocarnitine treatment (Figure 1C).

### Structural Diversity of the Acyl-Homocarnitine Family

The CrAT-dependent generation of acyl-homocarnitines and our prior finding of CPT1-dependent C16-homocarnitine formation suggested that a larger family of acyl-homocarnitines may exist. To determine the diversity and concentration dependence of acyl-homocarnitine formation, we conducted an *in vitro* isotope tracer study with ^13^C_3_-homocarnitine. Fetal bovine serum was used in the cell culture medium as the source of fatty acids. Hepatoma (Huh7) cells were prioritized due to their previously observed activity for C2 and C16 acylation of homocarnitine.^1^ In addition, hepatoma (HepG2) cells have shown inhibited fatty acid oxidation from δ-valerobetaine treatment.^2^ Given the heart’s high dependence on fatty acid oxidation,^17,18^ previous findings that δ-valerobetaine inhibits this process in cardiomyocytes,^19,20^ and previous detection of C2 and C16 acyl-homocarnitines in mouse heart,^1^ a cardiac cell line and mouse tissue were also studied.

The ^13^C_3_-isotope label and corresponding mass shift (Δ 3.0099) enabled clear distinction of acyl-homocarnitines from coeluting acyl-carnitine isomers. Huh7 cells were treated with ^13^C_3_-homocarnitine (0–30 μM) for 18 h, and cell extracts were analyzed with 10 min HILIC and C18 chromatography methods. Based upon existing knowledge of acyl-carnitine structures,^13–16^ theoretical *m*/*z* values for acyl-^13^C_3_-homocarnitines were calculated, and matches within ± 5 ppm were documented. Signals exclusively detected in treated cells were integrated to obtain relative abundances and were subjected to targeted MS^2^ (HCD 35%). A diagnostic ^13^C_3_-labeled nitrogen product ion at *m*/*z* 63.0908, along with at least two other homocarnitine product ions, including *m*/*z* 99.0441, *m*/*z* 117.0546, or *m*/*z* 161.1276 (*m*/*z* 158.1176 without ^13^C_3_)^1^ within ± 5 ppm, were used to assign identity as an acyl-^13^C_3_-homocarnitine.

Twelve acyl-^13^C_3_-homocarnitines met the treatment-dependence (Supporting Information 1, Figure S4) and MS^2^ product ion criteria (Supporting Information, Figures S5–16). Based on peak area, propionyl-^13^C_3_-homocarnitine (C3) (*m*/*z* 235.1648) was the most abundant (Figure 2A–B). After propionyl-^13^C_3_-homocarnitine, the relative intensities from highest to lowest included C2, C4, and C5 -homocarnitine (Figure 2C) followed by lower-abundance forms, including C5:1, C14, C6, C18:1, C8:1, C16, and C8 (Figure 2D). Lower-abundance, long-chain ^13^C_3_-acylhomocarnitines (e.g., C16) exhibited higher variability at 10 and 30 μM ^13^C_3_-homocarnitine treatment. This variability appeared to be biological in nature rather than from analytical or experimental variations. For this reason, tracer experiments were repeated across a broader concentration range, which yielded similar diversity in ^13^C_3_-acylhomocarnitine production (Supporting Information, Figure S17). Variability was low at 50 and 100 μM, whereas 15 μM produced variability comparable to that observed at 10 and 30 μM, suggesting that a transitional concentration range may exist in which long-chain acylation of homocarnitine occurs inconsistently in this cell type. Experiments with differentiated AC16 cardiomyocytes^8^ showed formation of six short-chain (C2–C6) acyl-homocarnitines with C2 as the most abundant (Supporting Information 1, Figure S18).

**Figure 2:**
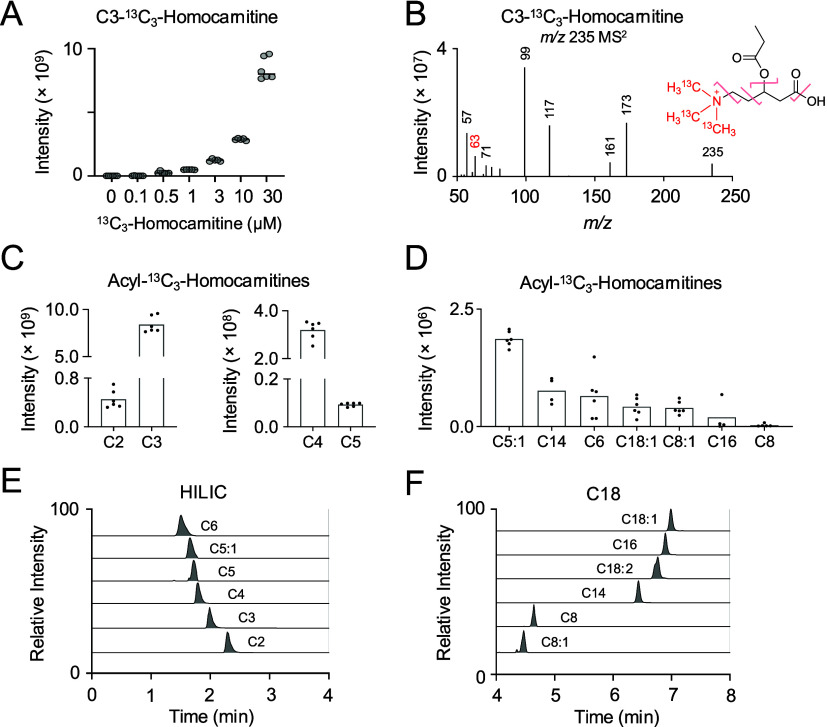
Isotope tracing with ^13^C_3_-homocarnitine enables discovery of a structurally diverse acyl-homocarnitine family. Human hepatoma Huh7 cells were treated with ^13^C_3_-homocarnitine at 0, 0.5, 1, 3, 10, or 30 μM for 18 h (*n* = 6 each). (A) Increase in peak area across concentrations for *m*/*z* 235.1468, matching annotation for a C3-^13^C_3_-homocarnitine. Dose–response data for the other acyl-^13^C_3_-homocarnitines are available (Supporting Information, Figure S4). (B) MS^2^ ion dissociation (HCD 35%) spectra of C3-^13^C_3_-homocarnitine (*m*/*z* 235.1648) show expected product ions including ^13^C_3_-labeled trimethyl-nitrogen (red; *m*/*z* 63.0908). Proposed sites of ion dissociation are indicated by red lines on the chemical structure. MS^2^ spectra with proposed product ion structures for all acyl-^13^C_3_-homocarnitines are available (Supporting Information, Figures S5–16). (C) At 30 μM ^13^C_3_-homocarnitine, peak areas of C2, C3, C4, and C5 acyl-^13^C_3_-homocarnitines. (D) Peak areas of the lower-abundant ^13^C_3_-acyl-homocarnitines, organized by abundance. One outlier removed for C14 and C16. C2 = *m*/*z* 221.1493, C3 = *m*/*z* 235.1648, C4 = *m*/*z* 249.1807, C5 = *m*/*z* 263.1965, C5:1 = *m*/*z* 261.1806, C6 = *m*/*z* 277.2118, C8:1 = *m*/*z* 303.2281, C8 = 305.2427, C14 = *m*/*z* 389.3371, C16 = *m*/*z* 417.3682, C18:1 = *m*/*z* 443.3842. C18:2 (*m*/*z* 441.3673) was detected in a subsequent study at 50 μM. This dose–response experiment was replicated with a wider concentration range (Supporting Information, Figure S17) and in a cardiac cell line (Supporting Information, Figure S18). (E) HILIC extracted ion chromatograms for short-chain acyl-^13^C_3_-homocarnitines and (F) C18 chromatography extracted ion chromatograms for medium and long-chain acyl-^13^C_3_-homocarnitines from Huh7 cells treated with 50 μM ^13^C_3_-homocarnitine. Each chromatogram was normalized to 100.

The structural diversity supports the involvement of multiple enzymes in acyl-homocarnitine metabolism. CrAT (EC:2.3.1.7) is most active with carnitine and C2–C10 fatty acids^10,21^ and likely contributed to the generation of C2–C5 acyl-homocarnitines and possibly the C6–C8 species (Figure 2C–D). CrOT (EC:2.3.1.137), which is most active with C6–12 chains,^22,23^ may have also contributed to C8, C8:1, C14 -homocarnitine formation (Figure 2D). Based on our previous demonstration of CPT1-dependent formation of C16-homocarnitine, it is reasonable to attribute the generation of C14-, C16-, C18:1-, and C18:2-homocarnitine to CPT1 (EC:2.3.1.21, which has three tissue-specific isoforms^24–26^ ) (Figure 2D). Given the wide variety of fatty acids in nature,^14^ other acyl-homocarnitines may exist at low concentrations. The observed relative abundance of acyl-homocarnitines may reflect enzyme substrate preferences or characteristics of our sample type. For instance, in human serum, C2-carnitine is most abundant^14^ whereas in the fetal bovine serum used to supplement our Huh7 cell culture, C3-carnitine was most abundant.

The precise impact of acyl-homocarnitine presence on fatty acid metabolism is not fully understood. Under conditions of elevated valerobetaine (e.g., impaired fatty acid oxidation and cardiometabolic disease^2–6^ ), a shift in acyl group handling from carnitine to homocarnitine could reduce the efficiency of mitochondrial fatty acid transport, β-oxidation, and CoA buffering. Carnitine and its precursor γ-butyrobetaine are evolutionarily conserved,^27,28^ abundant,^29,30^ and functionally optimized metabolites for enzymes within mammalian fatty acid metabolism,^31,32^ suggesting they are the preferred substrates for this pathway. Consistent with this, it has been shown that γ-butyrobetaine dioxygenase catalyzes hydroxylation of valerobetaine to homocarnitine ∼25-fold less efficiently than conversion of γ-butyrobetaine to carnitine;^33,34^ therefore, carnitine acyltransferases may also perform less efficiently with homocarnitine compared to carnitine.

### Ion Dissociation and Chromatographic Patterns of Acyl-^13^C_3_-Homocarnitines

MS^2^ spectra revealed product ions consistent with a neutral loss of the trimethylammonium group and/or loss of the acyl or acyl hydrocarbon chain, which enabled chain-length assignment for acyl-homocarnitines (Supporting Information, Figures S5–16). While the ion dissociation patterns and knowledge of fatty acid physiological abundance^13,14,29^ suggest mostly linear acyl chains, our mass spectrometry did not rule out the presence of branched-chain acyl-homocarnitines. In the case of C5-homocarnitine, valeric acid (straight-chain) is primarily microbiome-derived and abundant in ruminants,^35,36^ whereas isovaleric acid, a downstream leucine metabolite, is more abundant in humans.^14^ The use of bovine serum in human cell culture complicates this distinction. Additionally, the positions of unsaturation for C5:1, C8:1, C18:1, and C18:2 (Supporting Information, Figures S5–16) can be inferred because higher-energy collisional dissociation (HCD) often results in fragmentation adjacent to double bonds due to differential electron distribution.^37,38^ Based on the fragmentation behavior and natural abundance of oleic (C18:1) and linoleic (C18:2) acid,^29^ we inferred an ω-9 cis double bond for each and an additional ω-6 double bond for C18:2. Verification of double bonds will require future application of orthogonal methods^39^ such as epoxidation followed by MS^2^ ^37^ or fragmentation techniques such as OzID^40^ or UVPD.^41^

Chromatography of the acyl-^13^C_3_-homocarnitines showed expected retention time shifts based on the length of the acyl chain. In HILIC, retention decreased with increasing chain length for homocarnitine, C2, C3, C4, C5, C5:1, and C6 acyl-homocarnitines (Figure 2E). In C18 reverse-phase chromatography, retention increased with acyl chain length and less unsaturation for C8:1, C8, C14, C18:2, C16, and C18:1 acyl homocarnitines (Figure 2F). The elution order of the C18:1, C16, and C18:2 matched the elution order of synthetic C18:1, C16, and C18:2 acyl-carnitine standards. In these isotope tracer experiments, the ^13^C_3_ labeling enabled unambiguous distinction of acyl-homocarnitines from acyl-carnitines; however, endogenous metabolism studies require ion dissociation or chromatographic separation to distinguish isomers.

### Differentiation of Isomeric Acyl-Homocarnitines and Acyl-Carnitines by Ion Dissociation Patterns

Energy-resolved mass spectrometry (ERMS) was used to determine the collision energies at which diagnostic product ions form for three short-chain isomer pairs using collision-induced dissociation (CID) and higher-energy collisional dissociation (HCD). Relatively slow energy equilibration from CID yielded fewer but more quantifiable fragments, while HCD produced more fragments for isomer differentiation.^42^ Normalized CID or HCD collision energies (CEs) were increased in steps of 2 (10 to 62) for CrAT-generated C2-homocarnitine and C3-carnitine.

With CID, C2-homocarnitine produced four product ions (*m*/*z* 99.0437, 117.0544, 158.1175, *m*/*z* 159.0650) (Figure 3A), whereas C3-carnitine (*m*/*z* 218.1387) produced three (*m*/*z* 85.0281, 144.1017, 159.0650) (Figure 3B). The C2-homocarnitine product ions at *m*/*z* 99, 158, and 159 are analogous to C3-carnitine products with an additional methylene group in the first two cases (Supporting Information, Figure S19). These findings identified *m*/*z* 117 and 99 as diagnostic ions for C2-homocarnitine and *m*/*z* 85 and 144 as diagnostic ions for C3-carnitine. Although *m*/*z* 159 was the most abundant product ion for C2-homocarnitine, it was also formed from C3-carnitine. MS^3^ analysis showed C2-homocarnitine’s *m*/*z* 159 further dissociates into *m*/*z* 117 and 99, while C3-carnitine’s *m*/*z* 159 dissociates into *m*/*z* 85 (Supporting Information, Figure S20). Therefore, while *m*/*z* 159 may provide greater sensitivity for quantifying C2-homocarnitine under CID, its shared formation with carnitine limits selectivity without MS^3^ or chromatographic separation.

**Figure 3:**
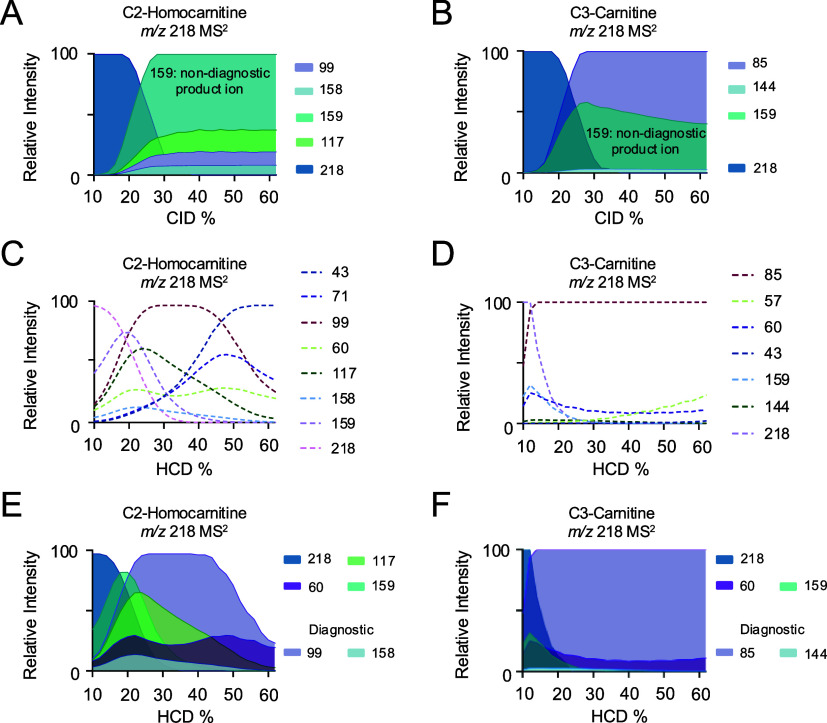
Comparison of ion dissociation patterns for isomers C2-homocarnitine and C3-carnitine (*m/z* 218.1387). Energy-resolved mass spectrometry (ERMS) for CrAT-generated C2-homocarnitine and C3-carnitine conducted by ramping normalized higher-energy dissociation (HCD) or collision-induced dissociation (CID) energies by steps of 2 (10–62) using HILIC coupled to a Thermo Scientific Orbitrap ID-X. ERMS breakdown curves are shown with relative intensities of product ions (relative intensity (%) = (intensity of ion/intensity of base peak ion) × 100). (A) Increasing CID % for C2-homocarnitine revealed 4 main MS^2^ product ions, including *m*/*z* 99.0437, 117.0544, 158.1175, and 159.0650 as the most intense. (B) Increasing CID % for C3-carnitine revealed 3 main MS^2^ product ions, including *m*/*z* 144.1017, 159.0650, and 85.0281 as the most intense. MS^3^ showed homocarnitine’s *m*/*z* 159 and carnitine’s *m*/*z* 159 are structurally different (Supporting Information, Figure S20). (C) Relative intensities of precursor and product ions for C2-homocarnitine formed by increasing HCD %. (D) Relative intensities of precursor and product ions for C3-carnitine formed by increasing HCD %. (E) Relative intensities of only the major product ions from HCD of C2-homocarnitine. (F) Relative intensities for C3-carnitine product ions that are the same (*m*/*z* 60, 159) or structurally analogous to isomeric C2-homocarnitine (*m*/*z* 99 and 158 for acyl-carnitine’s 85 and 144). Proposed structures and accurate masses for these product ions are available (Supporting Information, Figure S19). ERMS plots for isomers C3-homocarnitine/C4-carnitine (*m*/*z* 232.1551) and C4-homocarnitine/C5-carnitine (*m*/*z* 246.1700) are available (Supporting Information, Figures S21–22). Precursor degradation plots with CE_50_ values (Supporting Information, Figure S23) and recommended CE ranges for producing diagnostic ions are available (Table S1).

HCD produced broader dissociation profiles with product ions at *m*/*z* 43, 60, 71, 99, 117, 158, 159 for C2-homocarnitine (Figure 3C) and *m*/*z* 43, 57, 60, 85, 144, 159 for C3-carnitine (Figure 3D). Under HCD, C2-homocarnitine yielded several major, quantifiable product ions compared to C3-carnitine (Figure 3E–F). At HCD 24–36%, C2-homocarnitine spectra were dominated by *m*/*z* 99 followed closely by 117 (Figure 3E), whereas for C3-carnitine, *m*/*z* 85 became the predominant product ion beginning at 14% (Figure 3F). Thus, HCD produced the same differentiating product ions for both isomers as CID (*m*/*z* 117 and 85; minor *m*/*z* 158 and 144); however, the *m*/*z* 99 formed from C2-homocarnitine under HCD was both sensitive and specific, minimizing the need for MS^3^ or chromatographic separation.

C3-homocarnitine/C4-carnitine and C4-homocarnitine/C5-carnitine isomer pairs (Supporting Information, Figures S21–22) exhibited similar behaviors under both CID and HCD, with HCD being superior in generating a sensitive diagnostic ion at *m*/*z* 99 for homocarnitines, along with characteristic product ions at *m*/*z* 71 and 117. CID showed overlap of major product ions for acyl-homocarnitine/acyl-carnitine isomer pairs (*m*/*z* 159 for C3/C2; *m*/*z* 173 for C4/C3; and *m*/*z* 187 for C5/C4). In such cases, MS^3^ analysis or chromatographic separation is needed for quantification. Acyl-homocarnitines required higher HCD energies to achieve comparable dissociation to acyl-carnitines, while energy requirements were the same for isomers under CID (Supporting Information, Figure S23). Figure S23 summarizes the ERMS results with CE_50_ values.^43^ Recommended CE ranges for producing abundant, diagnostic product ions for each isomer pair are available (Table S1).

### Chromatographic Separation of Short-Chain Isomers

Limited separation based on hydrophobicity was obtained for short-chain acyl-homocarnitine and acyl-carnitine isomers on BEH amide HILIC (Figure 1B; Supporting Information, Figure S3) and C18 methods under common metabolomics run times (5–30 min), consistent with a marginally lower predicted logP value for C2-homocarnitine (XlogP3 = 0.7; CLogP = −3.92)^1^ compared to C3-carnitine (XlogP3 = 0.9; CLogP = −3.39).^44^ Topological polar surface area (tPSA = 66.4 Å) was identical for both isomers.^44,45^ We tested multiple gradients, buffers, temperatures, flow rates, and column chemistries and found that retention was markedly better on HILIC or zwitterionic stationary phase methods, as expected.^16^ A 40 min run time on a Waters BEH Amide HILIC column with ammonium acetate and 0.1% formic acid, produced some separation with C2-homocarnitine eluting first; however, baseline separation was not achieved. Increasing formic acid from 0.1 to 1% reversed the elution order of C2-homocarnitine and C3-carnitine, suggesting that ionic differences contribute to chromatographic behavior.

To test whether the reduced internal shielding of charges on homocarnitine permits stronger ionic interactions with a charged stationary phase, we used mixed-mode separation to leverage ionic and hydrophobic properties. Chromatographic separation of the isomer pairs was achieved with a Thermo Accucore solid core, unbonded silica HILIC column (2.1 mm × 50 mm, 2.6 μm), which exhibits stronger cation-exchange activity than amide or zwitterionic stationary phases.^46^ With the mobile phase buffered by 25 mM ammonium formate (pH 6.6), the silanol groups (p*K*_a_ of 4.7 ^47^) were mostly deprotonated (SiO^–^; ∼98.76%). Separation of C2-homocarnitine (p*K*_a_ of 3.80 ^45^) and C3-carnitine (p*K*_a_ of 3.02 ^44^) was achieved with a 10 min run time, which allowed for high-throughput analyses. Isomer pairs C4-homocarnitine/C5-carnitine, C3-homocarnitine/C4-carnitine, and C2-homocarntine/C3-carnitine were separated by 0.80, 0.85, and 0.86 min, respectively (Figure 4A). Related compounds, including homocarnitine, carnitine, γ-butyrobetaine, and δ-valerobetaine, eluted after the acylated species (Figure 4B). Evaluation of buffer concentrations from 1 to 40 mM showed that 25 mM provided the best peak shape and unquantifiable carryover after high concentration injections. Thus, under these separation conditions, electrostatic interactions govern isomer class separation while hydrophobicity determines elution order within an acyl chain series.

**Figure 4:**
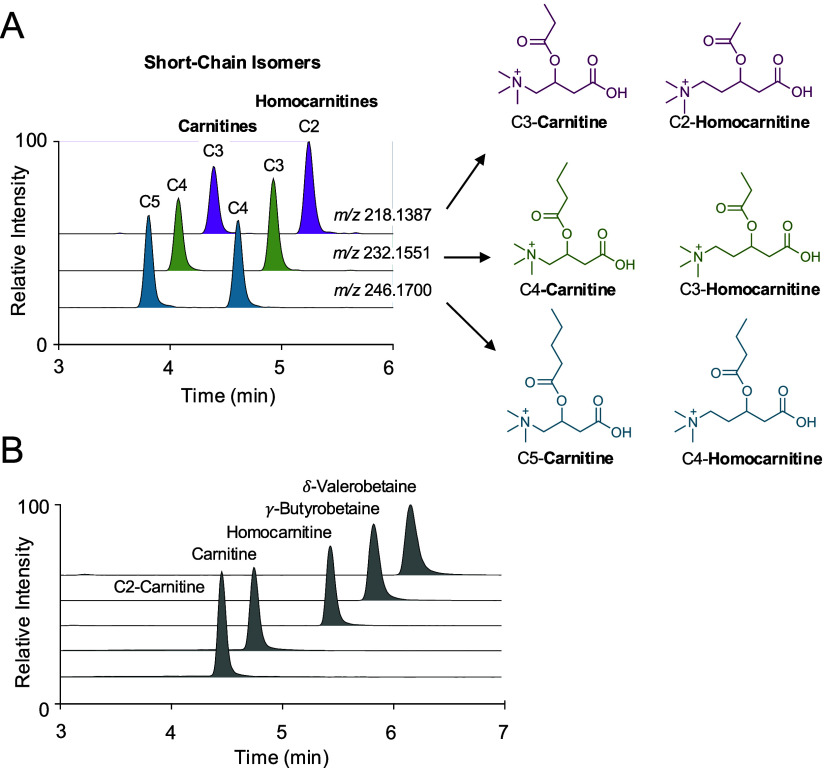
Chromatographic separation of short-chain acyl-homocarnitine and acyl-carnitine isomers. (A) Equimolar concentrations of CrAT-generated isomer pairs and synthetic C5-carnitine were analyzed on an optimized HILIC method. Extracted ion chromatogram showing all acyl-carnitines (C5, C4, C3) elute in a series (peaks on left) followed by isomeric acyl-homocarnitines (C4, C3, C2) (peaks on right). Structures of isomers on the right. C5-carnitine and C4-homocarnitine = *m*/*z* 246.1700. C4-carnitine and C3-homocarnitine = *m*/*z* 232.1551. C3-carnitine and C2-homocarnitine = *m*/*z* 218.1387. Chromatograms for each *m*/*z* were normalized to 100. (B) Equimolar concentrations of related species were analyzed on the optimized HILIC method. C2-carnitine = *m*/*z* 204.1230. Carnitine = *m*/*z* 162.1125. Homocarnitine = *m*/*z* 176.1281. γ-Butyrobetaine = *m*/*z* 146.1176. δ-Valerobetaine = *m*/*z* 160.1332.

### Acyl-Homocarnitine and Acyl-Carnitine Isomer Separation in Mouse Tissue

To test the optimized HILIC method for the separation of short-chain isomers from animal tissues, we analyzed heart tissue extracts from mice treated with or without δ-valerobetaine, the precursor for homocarnitine, for 12 weeks (Figure 5). To enrich compounds carrying a positive charge (i.e., acyl-homocarnitines), a sample preparation routine was developed that combined liquid-phase extraction followed by solid-phase mixed-mode cation-exchange (Supporting Information 1, Figure S24). This process was also expected to decrease sodium and potassium^48^ so that carnitines and betaines ionized exclusively as [M + H]^+^.

**Figure 5:**
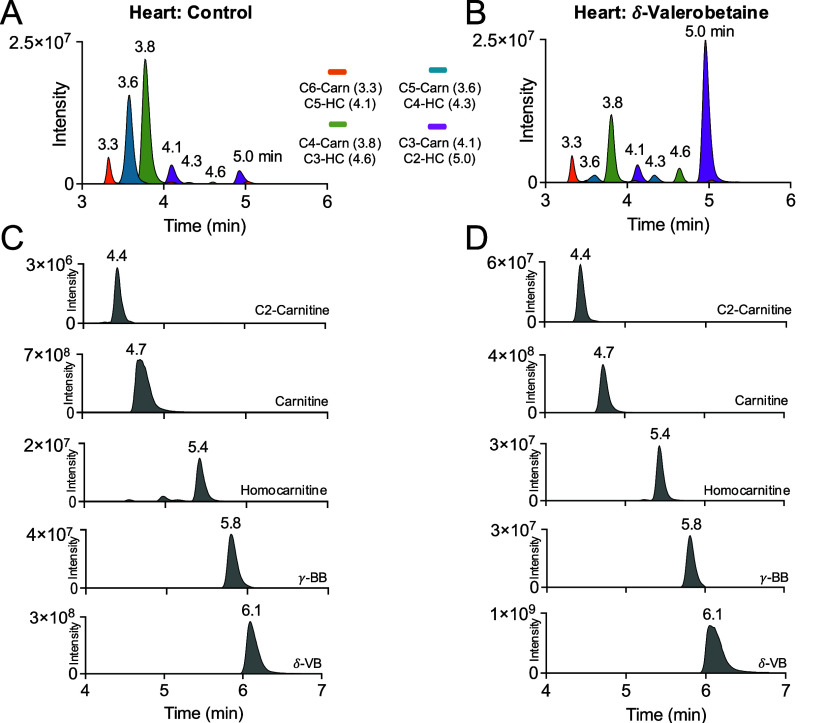
Chromatograms of acylated isomers, carnitines, and betaines from HILIC analysis of mouse heart. (A) HILIC isomer separation on a sample from mouse in control group (no δ-valerobetaine). C6-carnitine/C5-homocarnitine (orange; *m*/*z* 260.1856), C5-carnitine/C4-homocarnitine (blue; *m*/*z* 246.1700), C4-carnitine/C3-homocarnitine (green; *m*/*z* 232.1551), C3-carnitine/C2-homocarnitine (purple; *m*/*z* 218.1387). An overview of the sample preparation workflow is available (Supporting Information, Figure S24). Peak areas are reported in Supporting Information, Figure S25. (B) HILIC isomer separation on a sample from a mouse who had access to water treated with 1.62 mM δ-valerobetaine. (C) Extracted ion chromatograms of C2-carnitine, carnitine, homocarnitine, γ-butyrobetaine, δ-valerobetaine from a sample of a mouse in the control group or (D) δ-valerobetaine treatment group. A transition list is available (Table S2). Carn = carnitine. HC = homocarnitine.

Acyl-homocarnitines were detected in mouse heart from both groups and were increased in the δ-valerobetaine-treated mouse (Figure 5A–B; Supporting Information, Figure S25). Isomer separations were consistent between samples and matched those obtained from standards (0.72, 0.83, 0.83 min). A fourth isomer pair, C6-carnitine/C5-homocarnitine was also detected in both groups and separated by 0.76 min. The retention time of C6-carnitine was confirmed using a synthetic standard. The elution time of C5-homocarnitine matched the expected relative retention, and its MS^2^ ion dissociation pattern matched the spectra collected for C5-^13^C_3_-homocarnitine in the isotope tracing experiment (Figure 2; Supporting Information, Figure S8). From both mice, C2-carnitine, free carnitine, free homocarnitine, γ-butyrobetaine, and δ-valerobetaine were also measured (Figure 5C–D). Similar to the retention patterns from the acylated species, free homocarnitine and δ-valerobetaine eluted after their respective homologs, likely due to stronger ionic interactions with the stationary phase. A minor matrix effect was observed between the standard mix in methanol (Figure 4) and the tissue extracts (Figure 5) with a uniform retention time shift of 0.2 min across analytes. A transition list for all analytes and internal standards is available for download and can be imported directly into Skyline^12^ for analysis.

## Conclusions

The present studies elucidate a family of metabolites consisting of short-, medium-, and long-chain acyl-derivatives of homocarnitine and provide analytic methods for differentiation of these from isomeric acyl-carnitines. Acyl-homocarnitines were only recently detected upon discovery that the microbiome product, δ-valerobetaine, is hydroxylated to form homocarnitine. Lack of recognition and differentiation of acyl-homocarnitines may have contributed to mis-annotation as acyl-carnitines in metabolomics studies. Acyl-carnitines are often features of interest in clinical metabolomics studies, and they are of considerable importance in research on mitochondrial dysfunction, metabolic disease,^2,3,6,45^ and aging.^49,50^ The presently described methods provide diagnostic ions and liquid chromatography methods to distinguish acyl-homocarnitines from their acyl-carnitine isomers. This knowledge will enable improved understanding of microbiome-influenced fatty acid metabolism and mitochondrial dysfunction in disease and improve accurate metabolite identification in metabolomics studies. Related research will advance human health through improved resolution of acyl-homocarnitine and acyl-carnitine associations with human diseases.

## Supplementary Material







## Data Availability

This study is available at the NIH Common Fund’s National Metabolomics Data Repository (NMDR) website, the Metabolomics Workbench, https://www.metabolomicsworkbench.org, where it has been assigned Project ID PR002883. The data can be accessed directly via its Project DOI: 10.21228/M80 V8J. The Workbench is supported by NIH grant U2C-DK119886 and OT2-OD030544 grant.

## Notes

Additional data for enzyme, cell, ERMS, and tissue analysis, as well as MS^2^ spectra and proposed structures for acyl-^13^C_3_-homocarnitine product ions; generation of acyl-homocarnitine standards with carnitine acetyltransferase (CrAT) (Figure S1); positive control experiment for carnitine acetyltransferase (CrAT) generation of acylated standards (Figure S2); coelution of isomeric acyl-homocarnitines and acyl-carnitines (Figure S3); dose–response ^13^C_3_-homocarnitine tracer study in Huh7 cells (Figure S4); MS^2^ spectra and proposed structures of product ions for C2-homocarnitine (Figure S5); MS^2^ spectra and proposed structures of product ions for C3-homocarnitine (Figure S6); MS^2^ spectra and proposed structures of product ions for C4-homocarnitine (Figure S7); MS^2^ spectra and proposed structures of product ions for C5-homocarnitine (Figure S8); MS^2^ spectra and proposed structures of product ions for C5:1-homocarnitine (Figure S9); MS^2^ spectra and proposed structures of product ions for C6-homocarnitine (Figure S10); MS^2^ spectra and proposed structures of product ions for C8:1-homocarnitine (Figure S11); MS^2^ spectra and proposed structures of product ions for C8-homocarnitine (Figure S12); MS^2^ spectra and proposed structures of product ions for C14-homocarnitine (Figure S13); MS^2^ spectra and proposed structures of product ions for C16-homocarnitine (Figure S14); MS^2^ spectra and proposed structures of product ions for C18:1-homocarnitine (Figure S15); MS^2^ spectra and proposed structures of product ions for C18:2-homocarnitine (Figure S16); second dose–response ^13^C_3_-homocarnitine tracer study in Huh7 cells (Figure S17); dose–response ^13^C_3_-homocarnitine tracer study in AC16 cells (Figure S18); proposed structures for the major MS^2^ product ions of C2-homocarnitine and C3-carnitine, both *m*/*z* 218.1387 (Figure S19); MS^3^ spectra of *m*/*z* 159.0654, a nondifferentiating product ion of *m*/*z* 218.1387 (C2-homocarnitine or C3-carnitine) (Figure S20); energy-resolved mass spectrometry study of C3-homocarnitine and C4-carnitine isomers (Figure S21); energy-resolved mass spectrometry study of C4-homocarnitine and C5-carnitine isomers (Figure S22); CE_50_ values for isomers under CID or HCD (Figure S23); sample preparation workflow for isolation of homocarnitine, carnitines, and betaines (Figure S24); peak areas for acyl-homocarnitine and acyl-carnitine isomers in mouse heart (Figure S25) (PDF)
Recommended collision energies for isomers (Table S1) (XLSX)
Transition list (Table S2) (XLSX)

## Abbreviations and nomenclature

ACN: acetonitrile
CID: collision-induced dissociation
C#: number of carbons on acyl group
CrAT: carnitine acetyltransferase
CrOT: carnitine octanoyltransferase
CPT: carnitine palmitoyltransferase
ERMS: energy-resolved mass spectrometry
FBS: fetal bovine serum
HCD: higher-energy collisional dissociation
HILIC: hydrophilic interaction liquid chromatography
*m*/*z*: mass-to-charge ratio
