# Supplementary material for: Structural Diversity and Analytical Characterization of Acylhomocarnitines
Source: J Proteome Res. 2026 May 13;25(6):3054–65. doi: 10.1021/acs.jproteome.5c01255 (PMC13248012; doi:10.1021/acs.jproteome.5c01255)
Supplement: Supplementary file 1 [file pr5c01255_si_001.pdf]

Supporting Information

Structural diversity and analytical characterization of acylhomocarnitines

Jaclyn Weinberg[1], William J. Crandall[1], Zachery Ryan Jarrell[1], Gahyun Lim[1], Ken Liu[2], Ho-Young Lee[1], Shakshi Patel[3], Camilo Anthony Gacasan[4], Young-Mi Go[1], Dean P. Jones[1]*

Affiliations:
[1]Division of Pulmonary, Allergy, Critical Care and Sleep Medicine, Department of Medicine, Emory University School of Medicine, Atlanta, GA
[2]Department of Chemistry, Emory University, Atlanta, GA
[3]Department of Biochemistry, Department of Medicine, Emory University, Atlanta, GA
[4]Division of Gastroenterology, Hepatology, and Nutrition, Department of Medicine, Emory University, Atlanta, GA
*Corresponding Author (dpjones@emory.edu)

List of contents

Figure S1. Generation of acyl-homocarnitine standards with carnitine acetyltransferase (CrAT).
Figure S2. Positive control experiment for carnitine acetyltransferase (CrAT) generation of acylated standards.
Figure S3. Coelution of isomeric acyl-homocarnitines and acyl-carnitines.
Figure S4. Dose-response $^{13}C_3$-homocarnitine tracer study in Huh7 cells.
Figure S5. MS^2 spectra and proposed structures of product ions for C2-homocarnitine.
Figure S6. MS^2 spectra and proposed structures of product ions for C3-homocarnitine.
Figure S7. MS^2 spectra and proposed structures of product ions for C4-homocarnitine.
Figure S8. MS^2 spectra and proposed structures of product ions for C5-homocarnitine.
Figure S9. MS^2 spectra and proposed structures of product ions for C5:1-homocarnitine.
Figure S10. MS^2 spectra and proposed structures of product ions for C6-homocarnitine.
Figure S11. MS^2 spectra and proposed structures of product ions for C8:1-homocarnitine.
Figure S12. MS^2 spectra and proposed structures of product ions for C8-homocarnitine.
Figure S13. MS^2 spectra and proposed structures of product ions for C14-homocarnitine.
Figure S14. MS^2 spectra and proposed structures of product ions for C16-homocarnitine.
Figure S15. MS^2 spectra and proposed structures of product ions for C18:1-homocarnitine.
Figure S16. MS^2 spectra and proposed structures of product ions for C18:2-homocarnitine
Figure S17. Second dose-response $^{13}C_3$-homocarnitine tracer study in Huh7 cells.
Figure S18. Dose-response $^{13}C_3$-homocarnitine tracer study in AC16 cells.
Figure S19. Proposed structures for the major MS^2 product ions of C2-homocarnitine and C3-carnitine, both *m/z* 218.1387.
Figure S20. MS^3 spectra of *m/z* 159.0654, a non-differentiating product ion of *m/z* 218.1387 (C2-homocarnitine or C3-carnitine).
Figure S21. Energy-resolved mass spectrometry study of C3-homocarnitine and C4-carnitine isomers.
Figure S22. Energy-resolved mass spectrometry study of C4-homocarnitine and C5-carnitine isomers.
Figure S23. CE_{50} values for isomers under CID or HCD.
Figure S24. Sample preparation workflow for isolation of homocarnitine, carnitines, and betaines.
Figure S25. Peak areas for acyl-homocarnitine and acyl-carnitine isomers in mouse heart.

Supporting Information: Figure S1

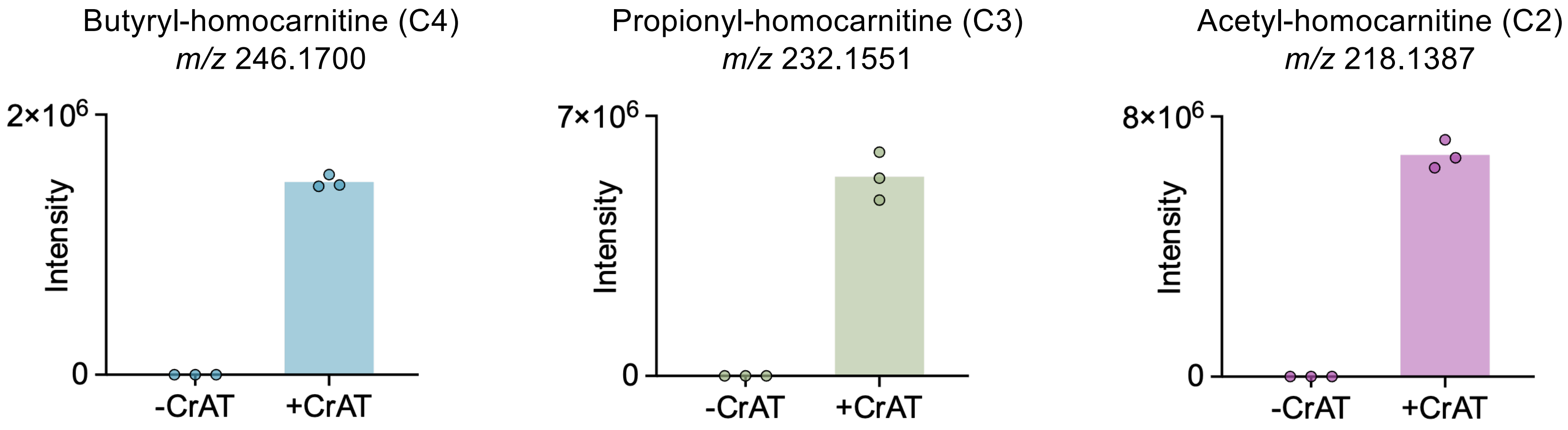

Figure S1. Generation of acyl-homocarnitine standards with carnitine acetyltransferase (CrAT). Homocarnitine and butyryl-CoA (C4), propionyl-CoA (C3), or acetyl-CoA (C2) were incubated +/- CrAT for 30 min at 37 °C and extracts were analyzed by HILIC/ESI+ (n = 3 each).

Supporting Information: Figure S2

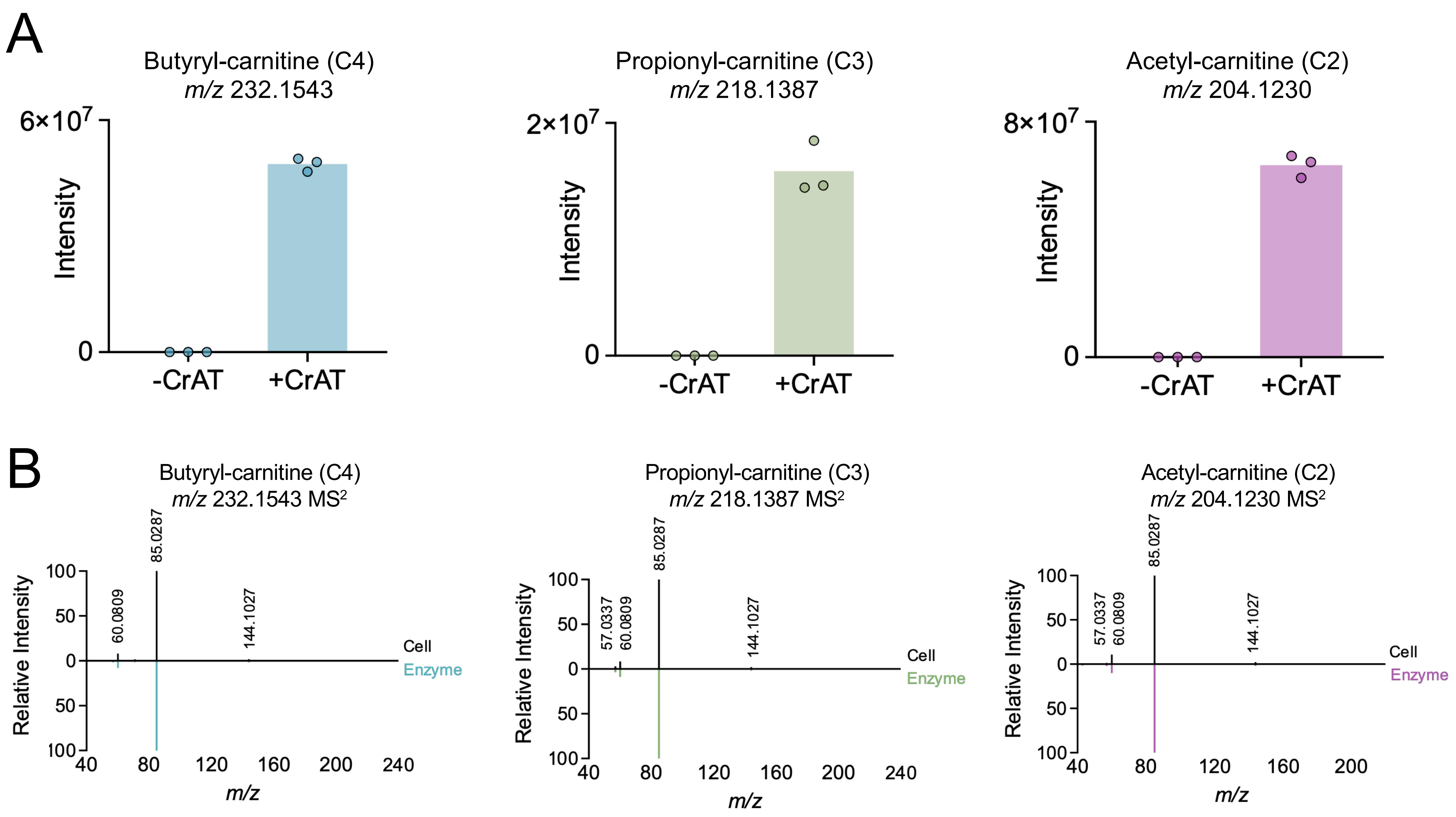

Figure S2. Positive control experiment for carnitine acetyltransferase (CrAT) generation of acylated standards. A. Carnitine and either butyryl-CoA (C4), propionyl-CoA (C3), or acetyl-CoA (C2) were incubated +/- CrAT for 30 min at 37 °C and extracts were analyzed by HILIC/ESI+ (n = 3 each). **B.** Mirror spectra comparing MS^2 ion dissociation (HCD 35%) of endogenous acyl-carnitines in untreated Huh7 cells (top) and enzyme generated standards (bottom).

Supporting Information: Figure S3

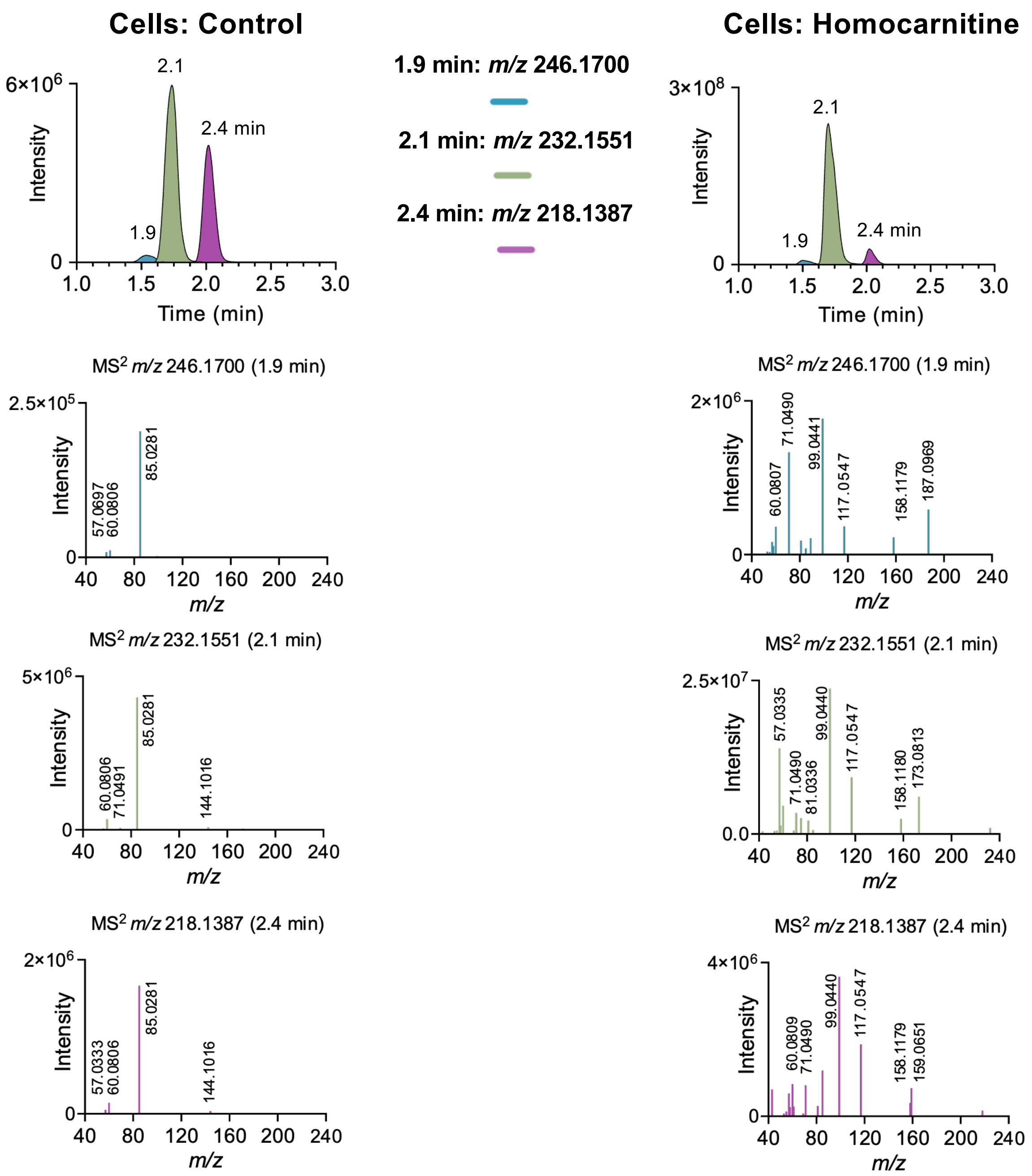

Figure S3. Coelution of isomeric acyl-homocarnitines and acyl-carnitines. Left: Huh7 cells treated with vehicle show mostly diagnostic ions for C5, C4, and C3 -carnitine. Right: Huh7 cells treated with 50 µM homocarnitine for 18 h show more ions suspected to be from C4, C3, and C2 -homocarnitine. All MS^2 spectra collected with HCD 35%.

Supporting Information: Figure S4

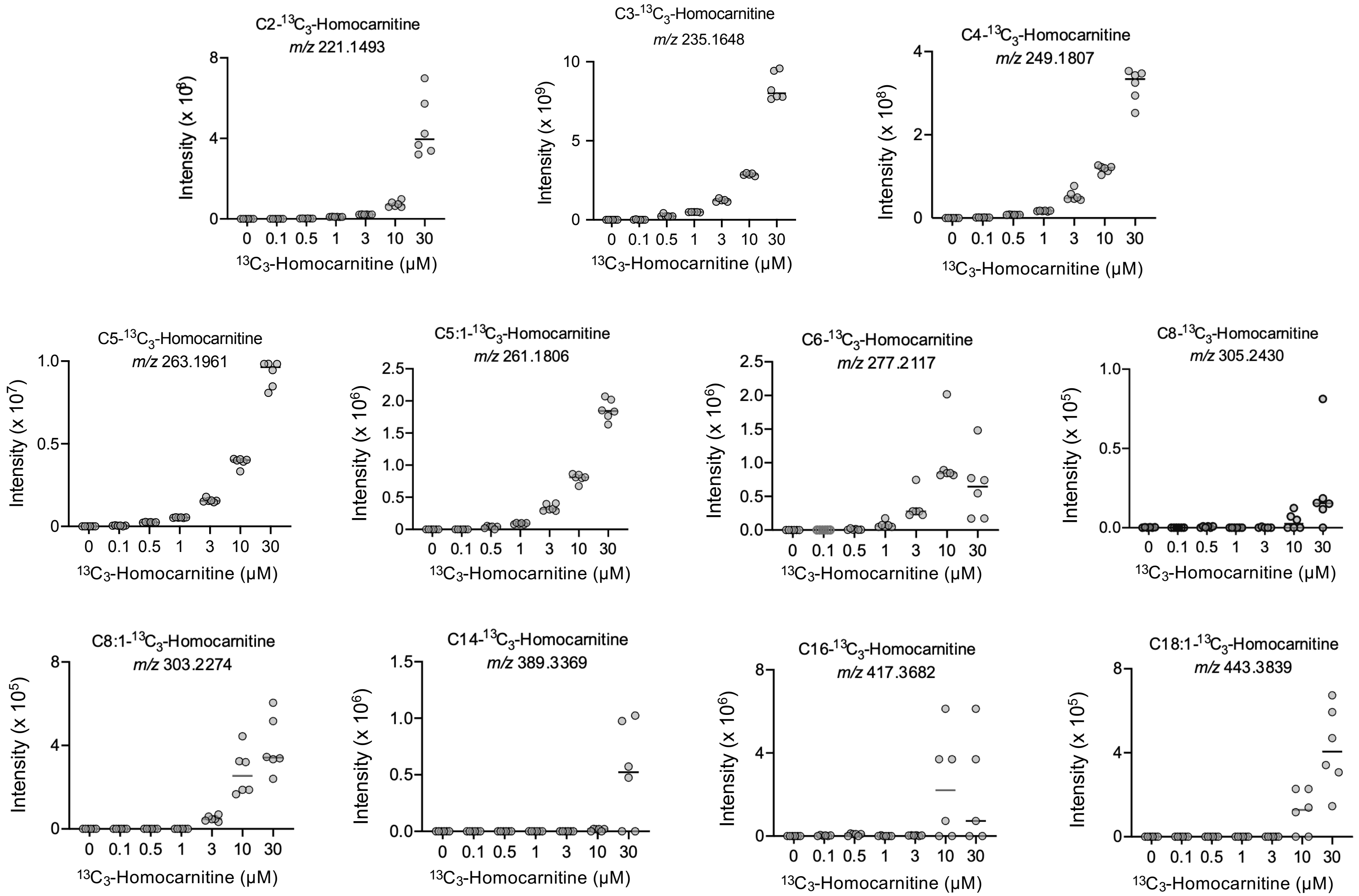

Figure S4. Dose-response $^{13}C_3$-homocarnitine tracer study in Huh7 cells. Cells were treated with $^{13}C_3$-homocarnitine for 16 h (N=6 for each, some replicates were non-detects). Signals matching a theoretical *m/z* for an acyl-$^{13}C_3$-homocarnitine were documented. Annotation required an increase with treatment, a MS^2 product ion at *m/z* 63.0809 representing the isotopically labeled trimethyl nitrogen, and at least two other characteristic acyl-homocarnitine product ions (*m/z* 99.0441, 117.0546, 158.1176 (161.1276 with $^{13}C_3$)).

Supporting Information: Figure S5

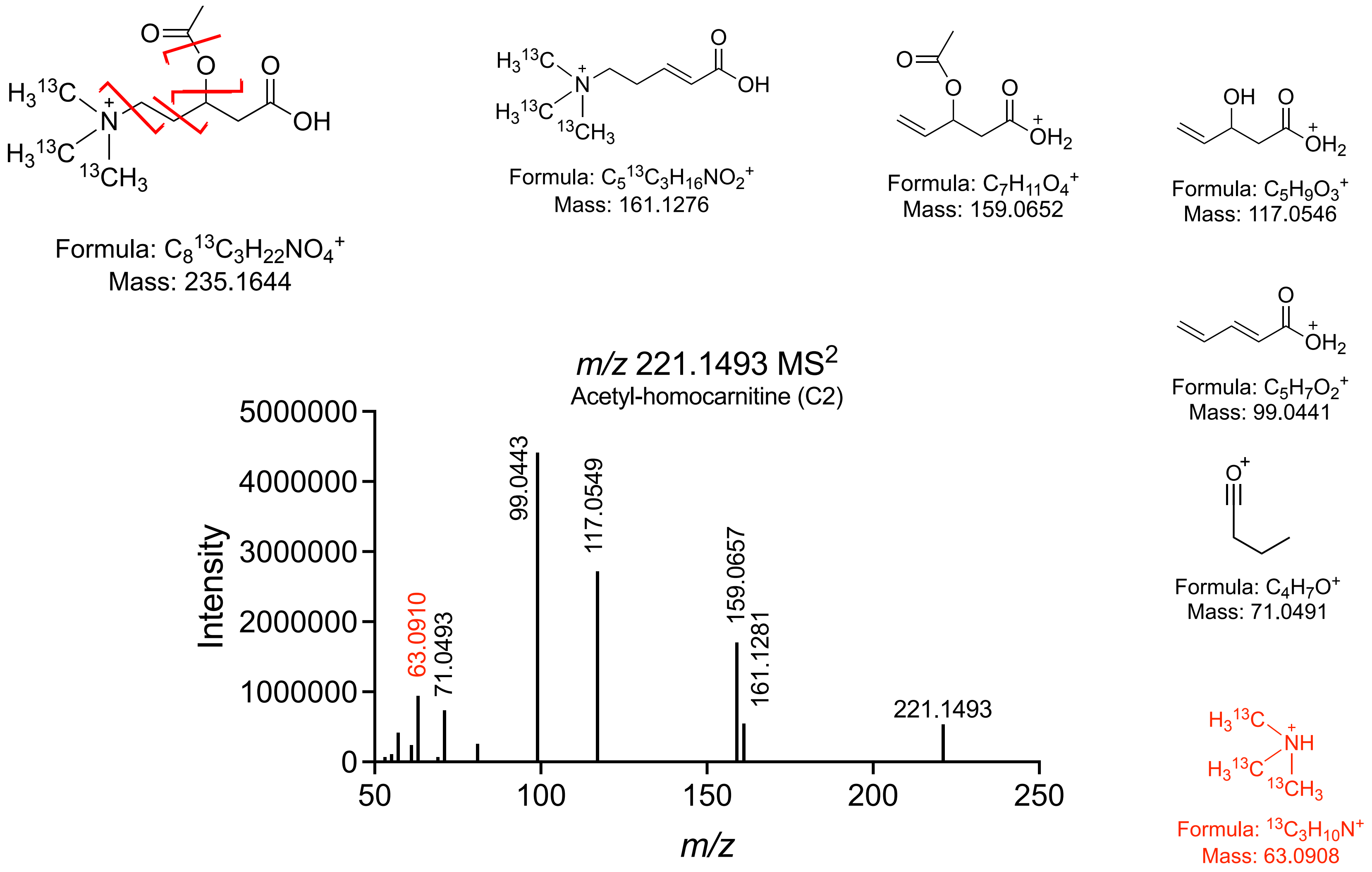

Figure S5. MS² spectra and proposed structures of product ions for C2-homocarnitine. HILIC/ESI+ with MS² at HCD 35%.

Supporting Information: Figure S6

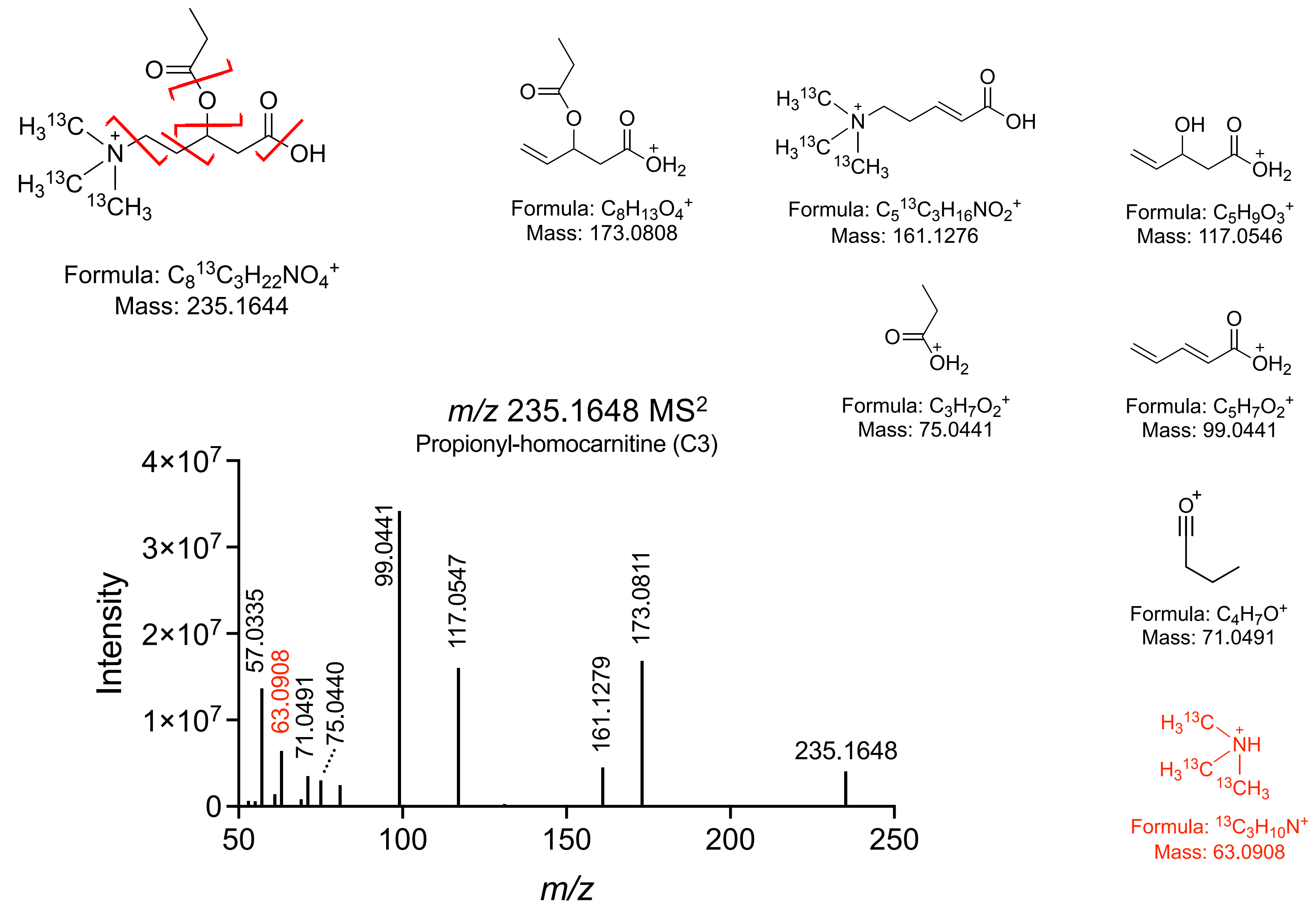

Figure S6. MS² spectra and proposed structures of product ions for C3-homocarnitine. HILIC/ESI+ with MS² at HCD 35%.

Supporting Information: Figure S7

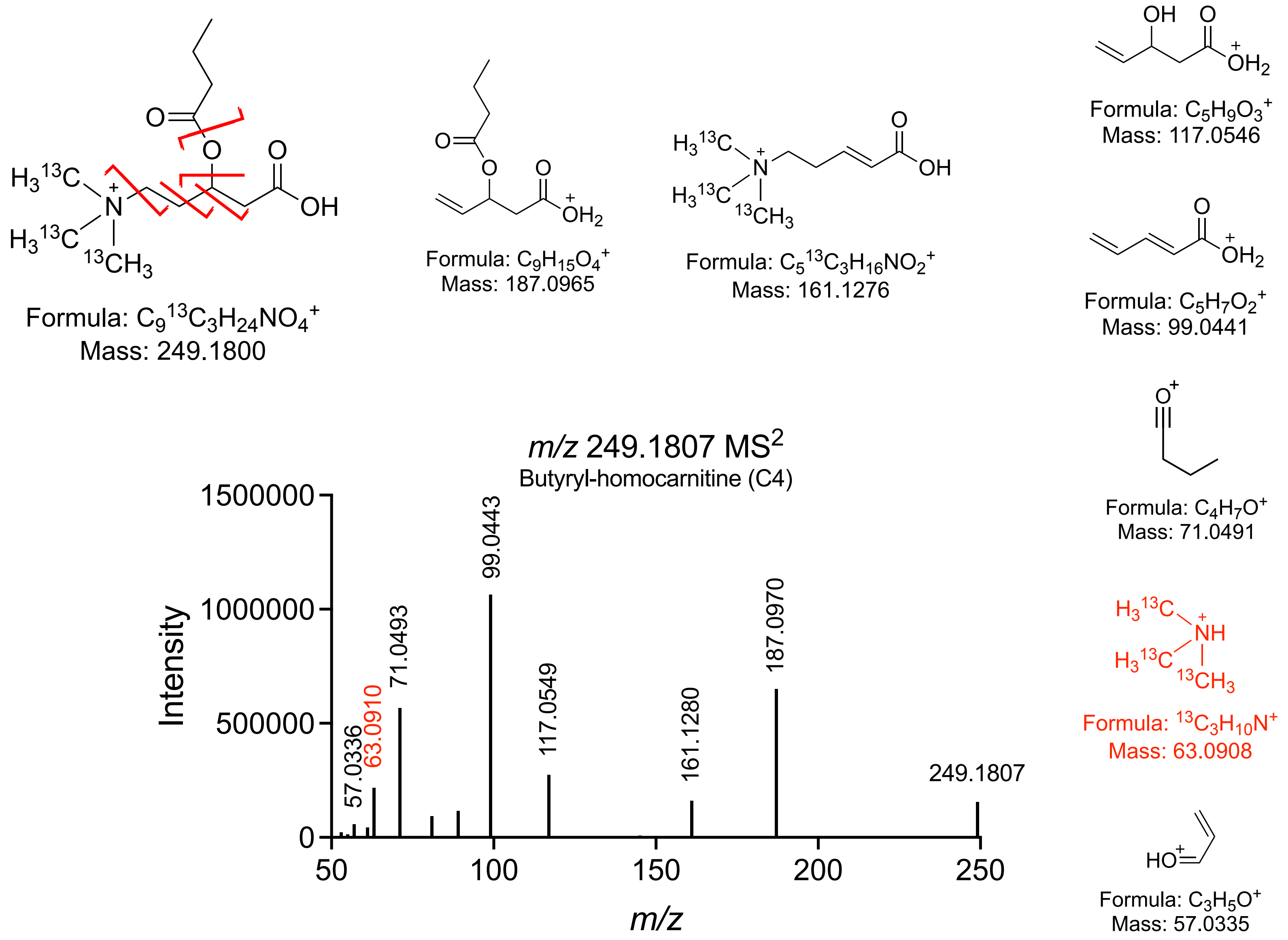

Figure S7. MS² spectra and proposed structures of product ions for C4-homocarnitine. HILIC/ESI+ with MS² at HCD 35%.

Supporting Information: Figure S8

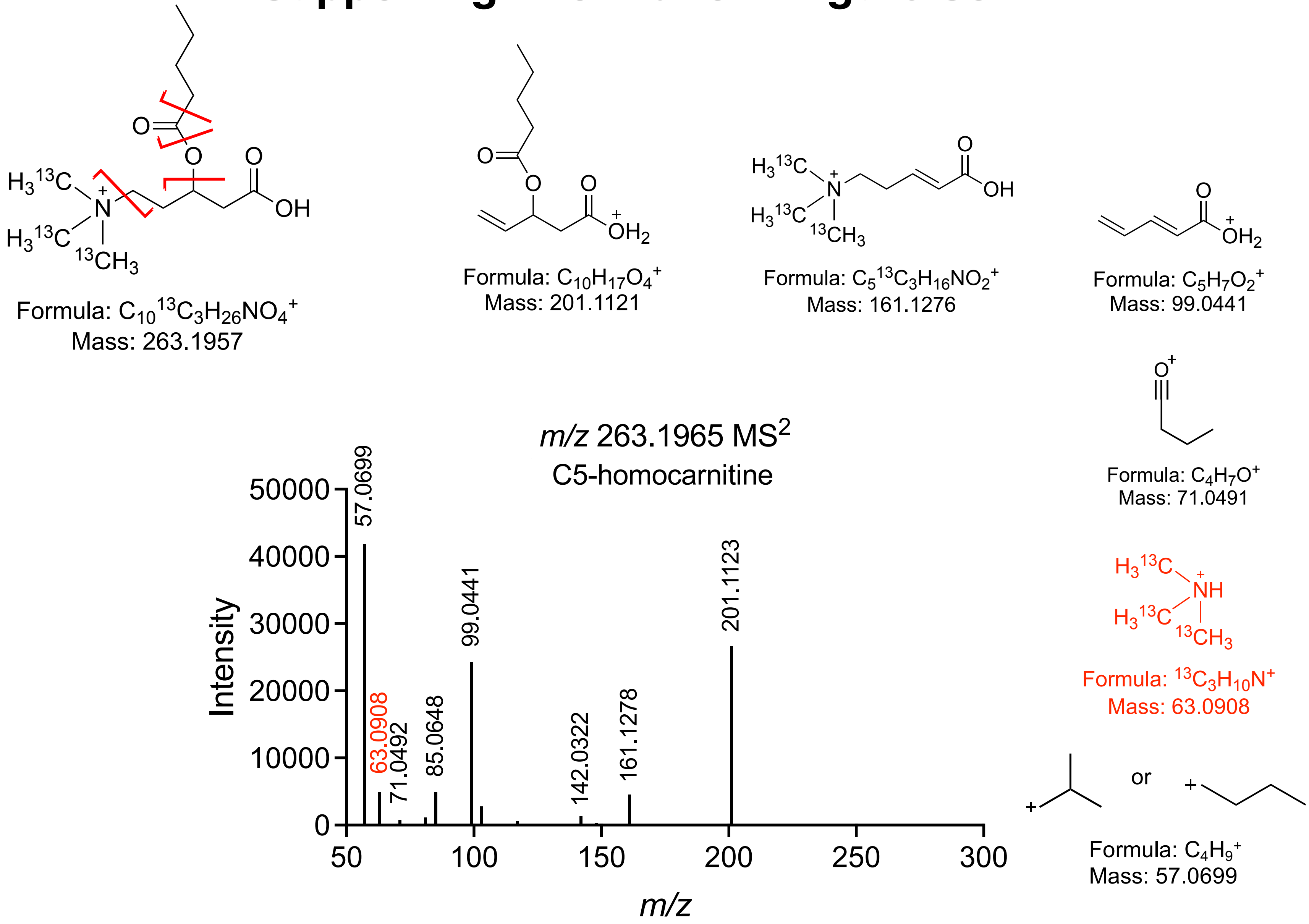

Figure S8. MS^2 spectra and proposed structures of product ions for C5-homocarnitine. HILIC/ESI+ with MS^2 at HCD 35%. Note: C5 could be straight chain (valeryl) or branched (isovaleryl or 2-methyl-butyryl). Isovaleryl-carnitine is typically most abundant in mammals as isovaleric acid is a breakdown product of leucine metabolism (Minkler et al., 2017).

Supporting Information: Figure S9

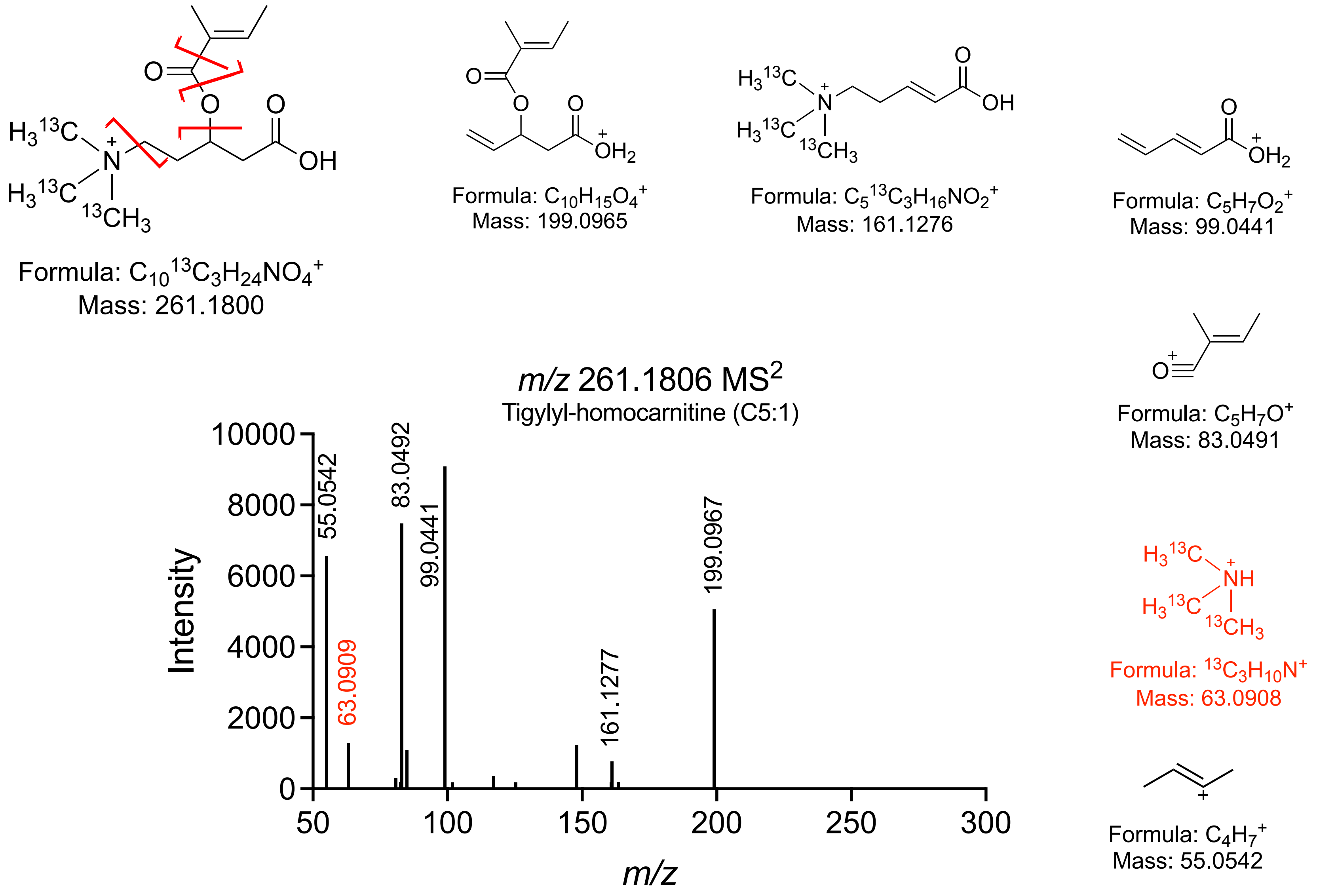

Figure S9. MS^2 spectra and proposed structures of product ions for C5:1-homocarnitine. HILIC/ESI+ with MS^2 at HCD 35%. Note: C5:1 could be straight chain or branched. Tigylyl (branched) is more likely as it is a breakdown product of isoleucine metabolism (Mandal et al., 2025).

Supporting Information: Figure S10

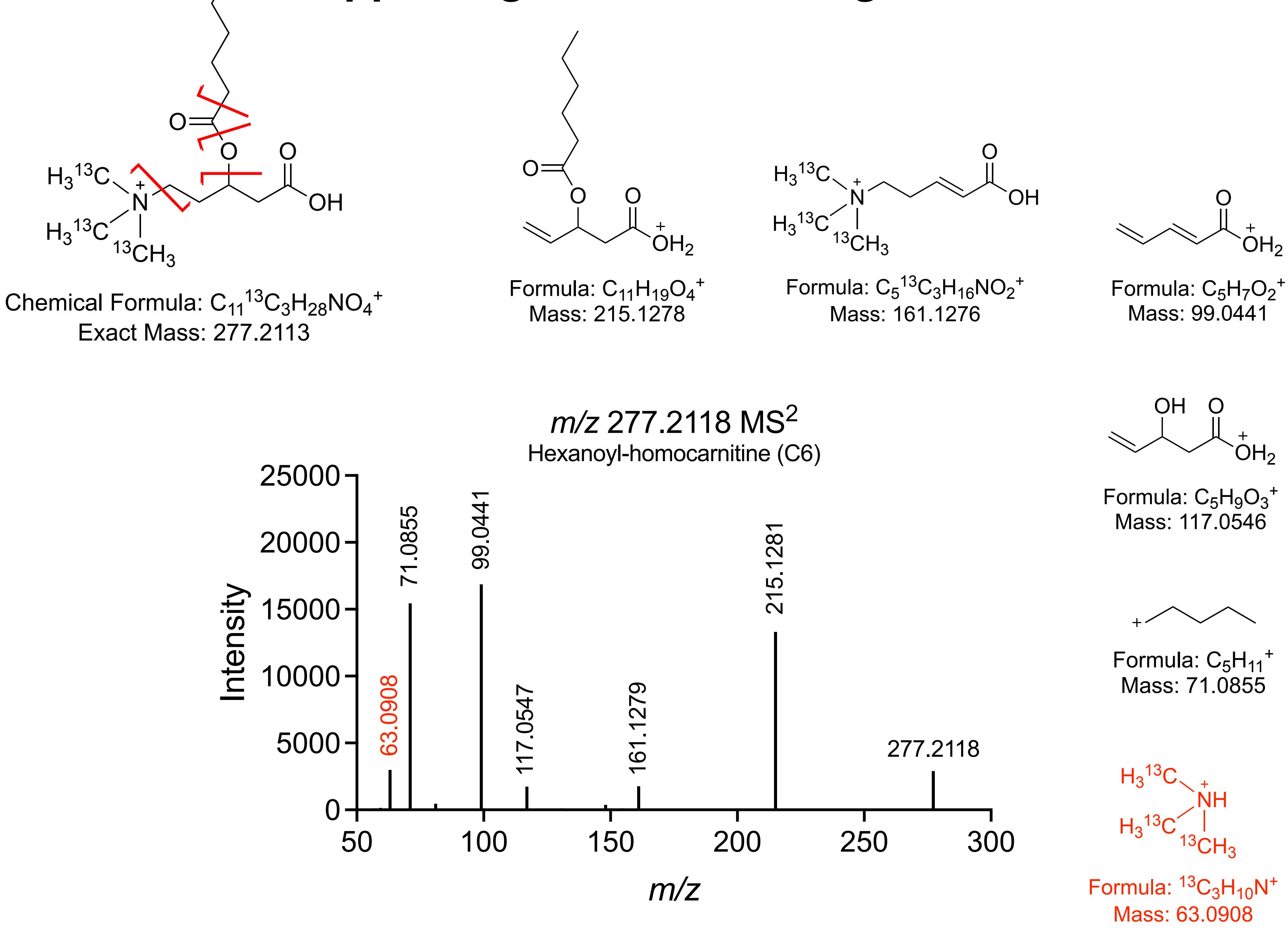

Figure S10. MS² spectra and proposed structures of product ions for C6-homocarnitine. HILIC/ESI+ with MS² at HCD 35%.

Supporting Information: Figure S11

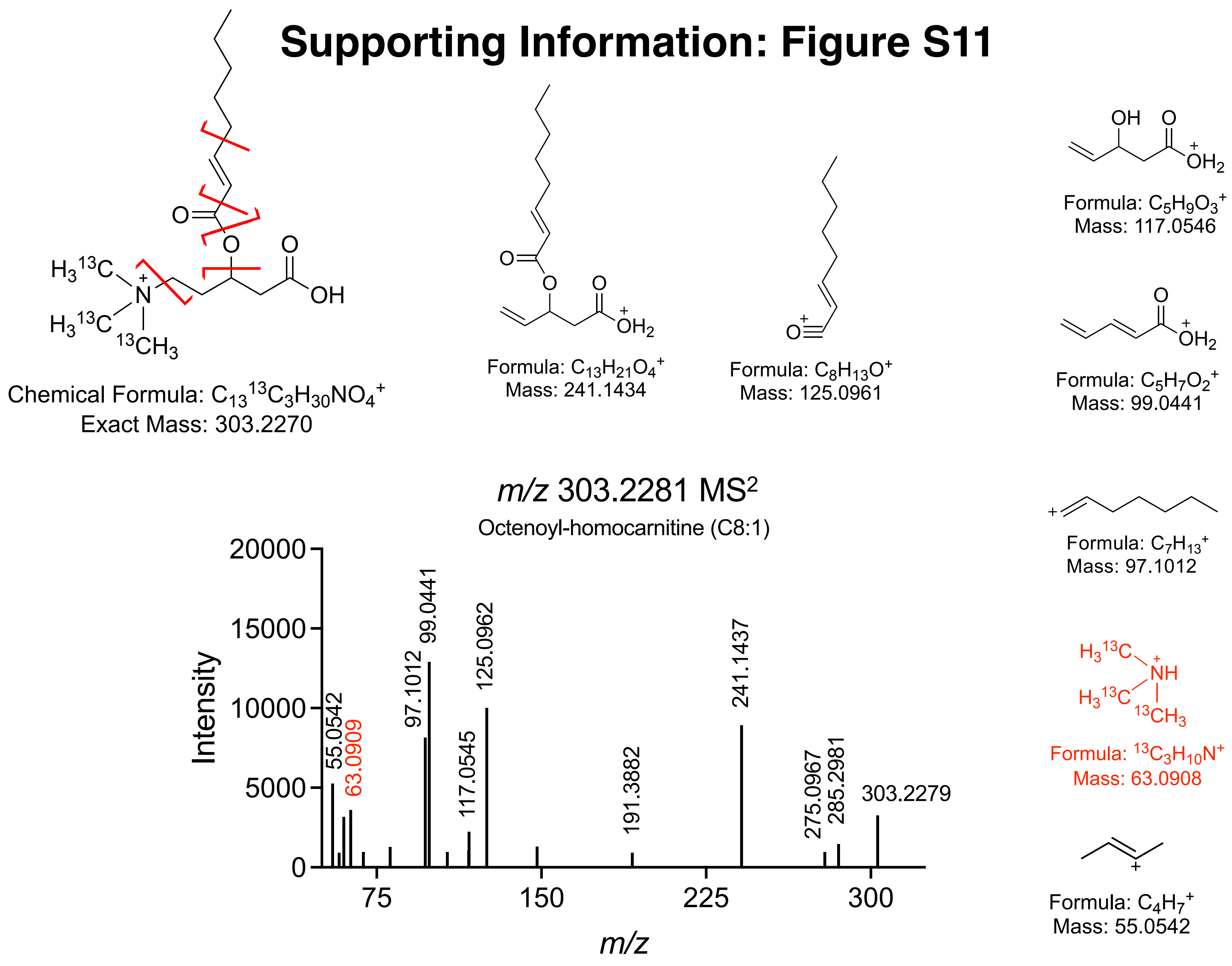

Figure S11. MS² spectra and proposed structures of product ions for C8:1-homocarnitine. HILIC/ESI+ with MS² at HCD 35%.

Supporting Information: Figure S12

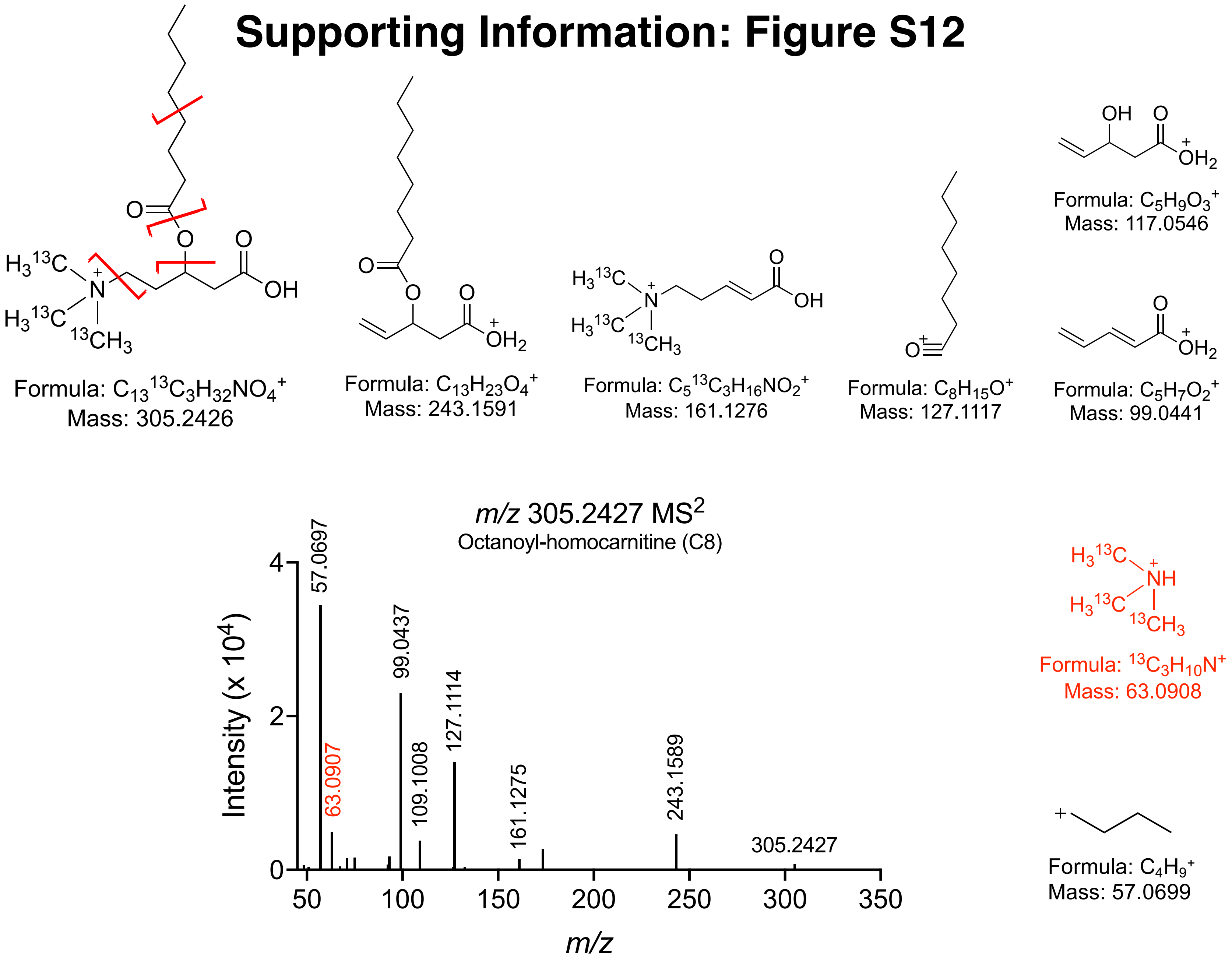

Figure S12. MS² spectra and proposed structures of product ions for C8-homocarnitine. HILIC/ESI+ with MS² at HCD 35%.

Supporting Information: Figure S13

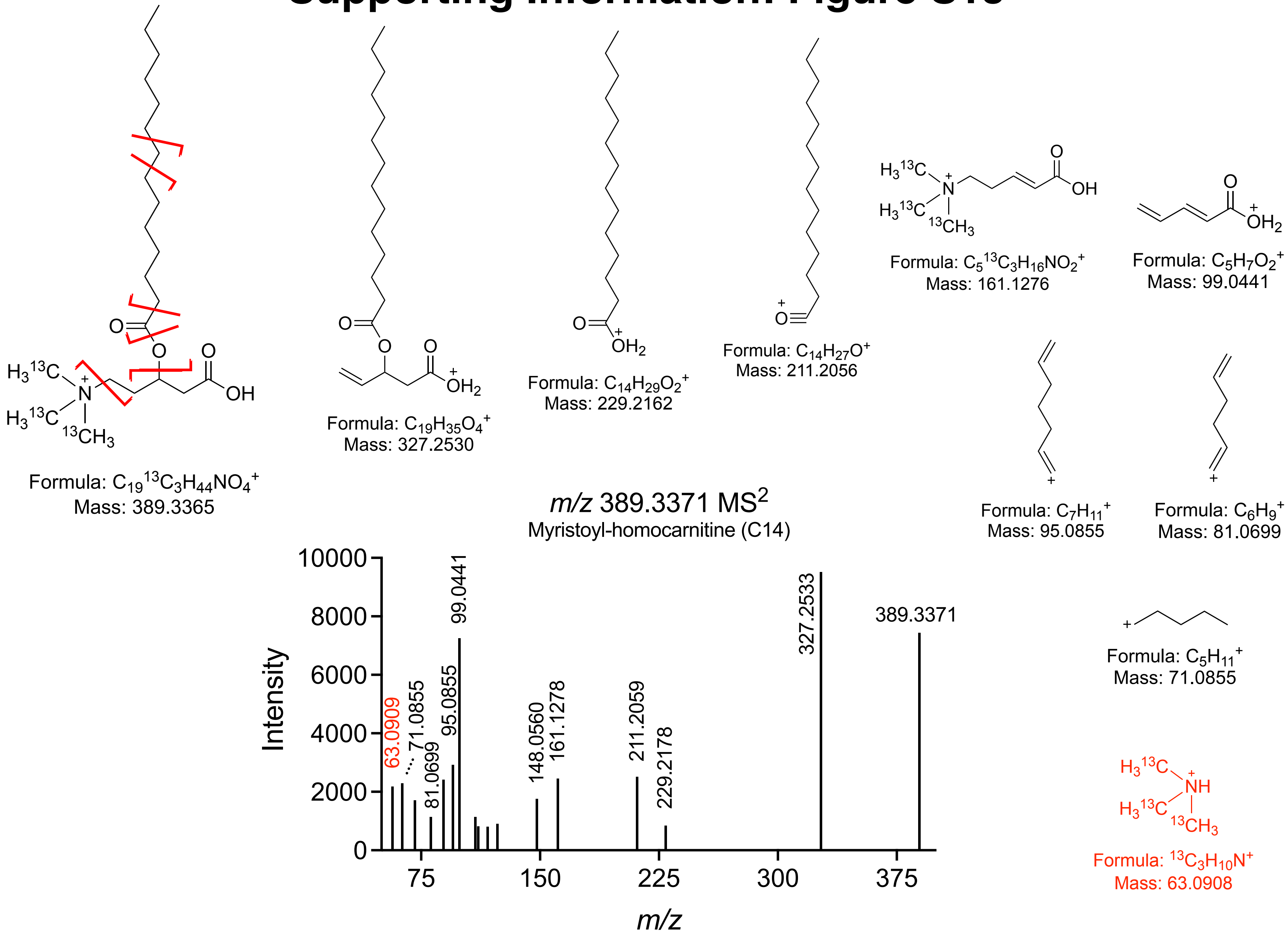

Figure S13. MS² spectra and proposed structures of product ions for C14-homocarnitine. HILIC/ESI+ with MS² at HCD 35%.

Supporting Information: Figure S14

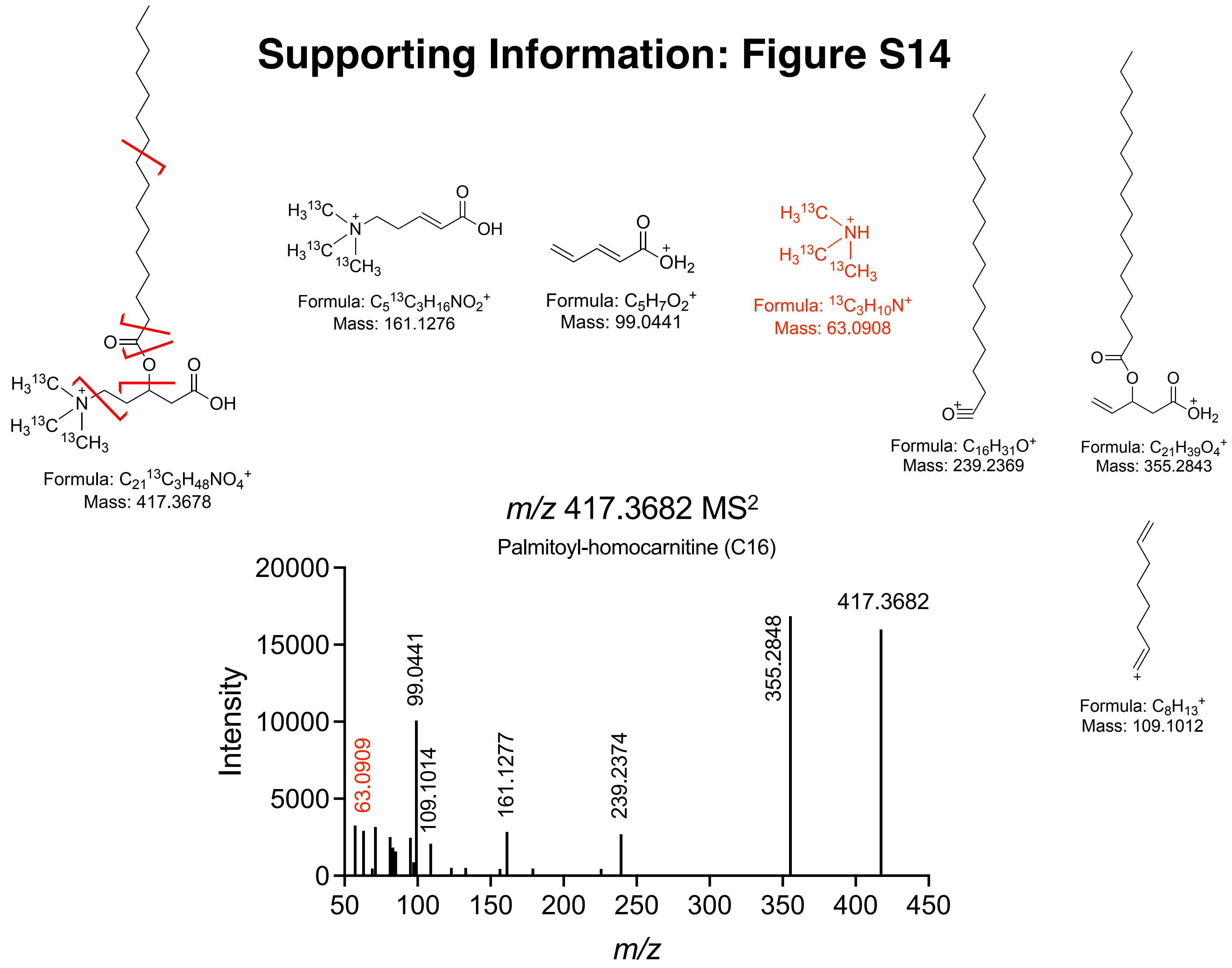

Figure S14. MS^2 spectra and proposed structures of product ions for C16-homocarnitine. HILIC/ESI+ with MS^2 at HCD 35%.

Supporting Information: Figure S15

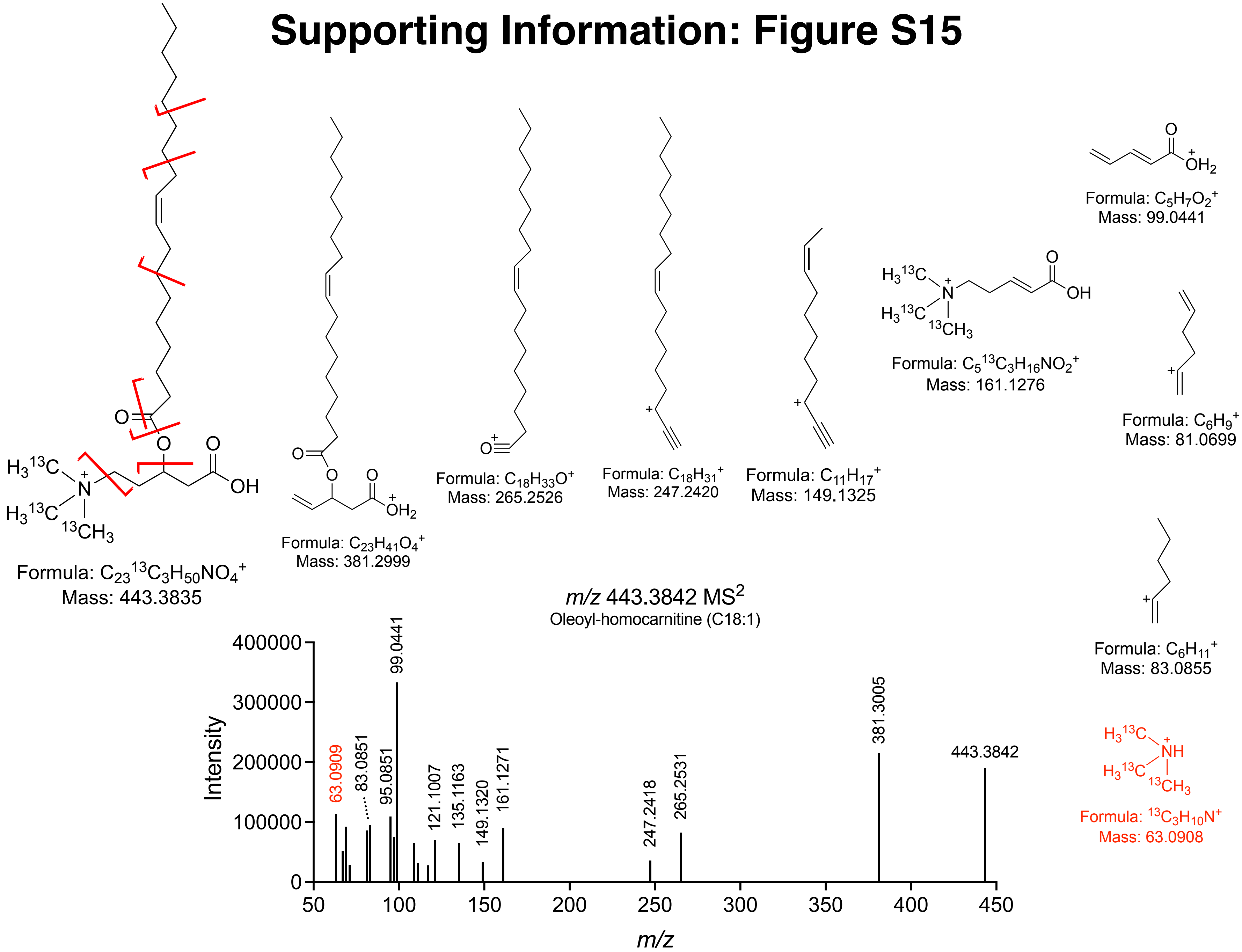

Figure S15. MS² spectra and proposed structures of product ions for C18:1-homocarnitine. HILIC/ESI+ with MS² at HCD 35%.

Supporting Information: Figure S16

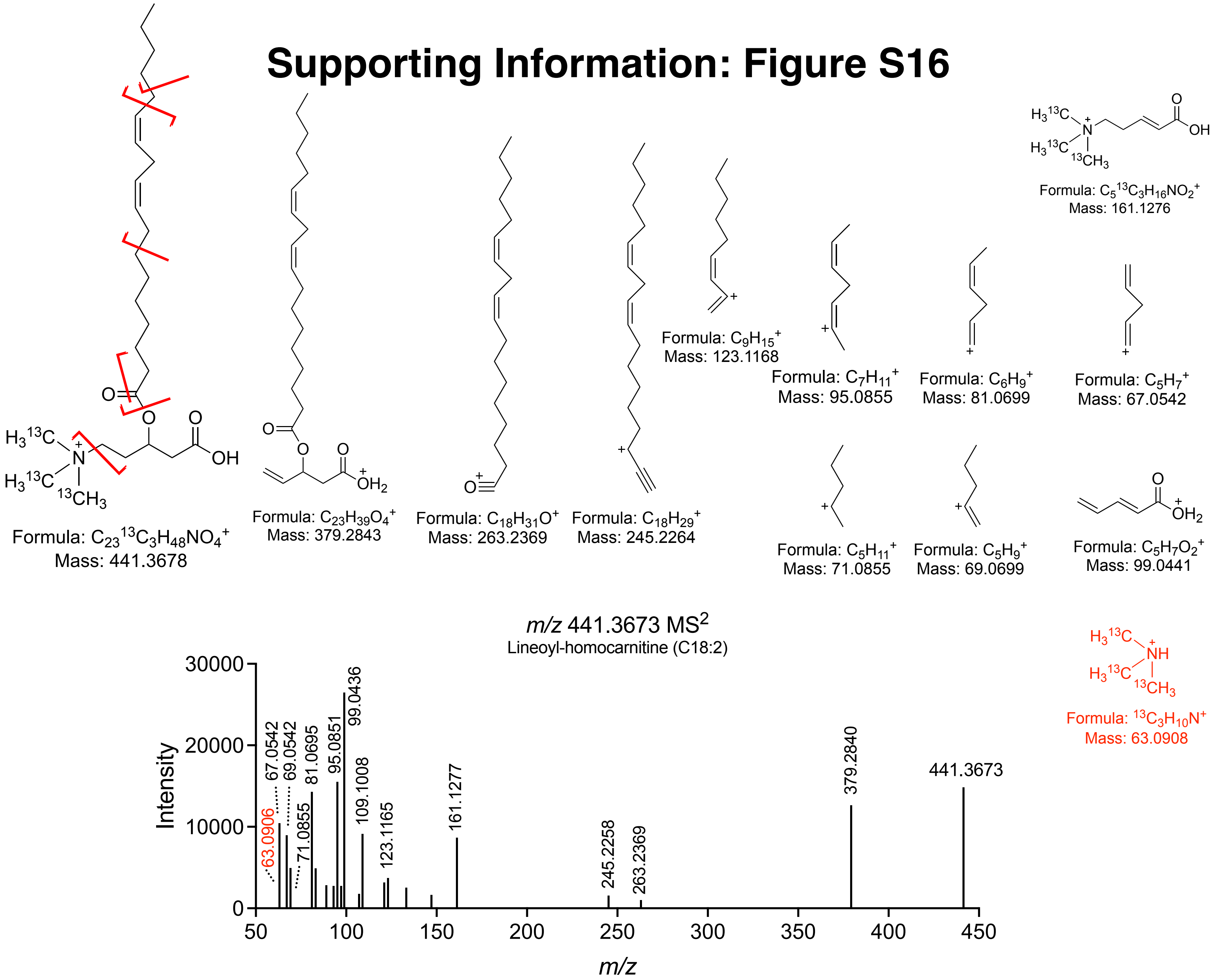

Figure S16. MS² spectra and proposed structures of product ions for C18:2-homocarnitine. HILIC/ESI+ with MS² at HCD 35%.

Supporting Information: Figure S17

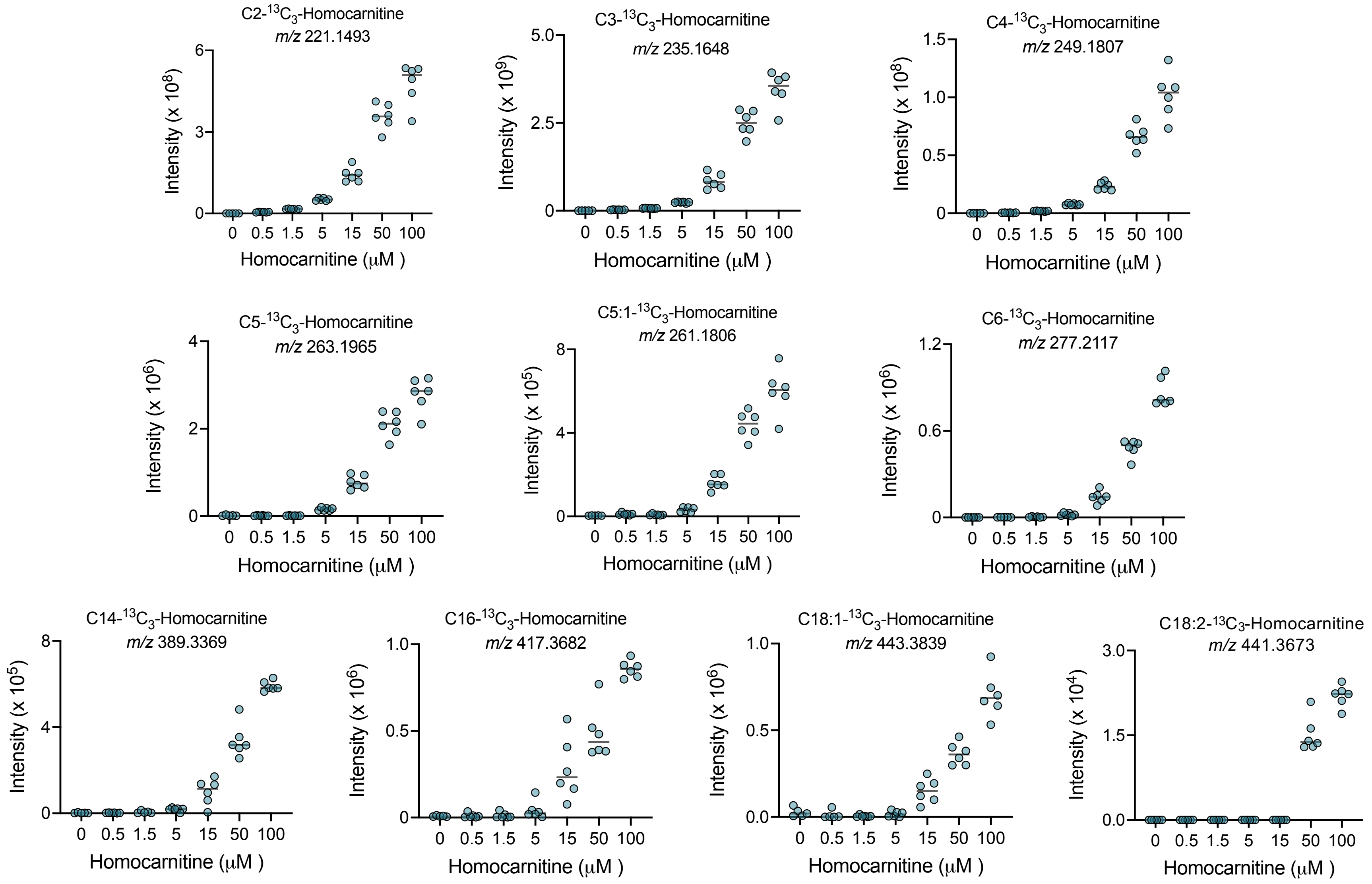

Figure S17. Second dose-response $^{13}C_3$-homocarnitine tracer study in Huh7 cells. Cells were treated with 0, 0.5, 1.5, 5, 15, 50 and 100 µM $^{13}C_3$-homocarnitine for 18 h (N=6 each). Signals matching a theoretical *m/z* for an acyl-$^{13}C_3$-homocarnitine. Annotation required an increase with treatment, a MS^2 product ion at *m/z* 63.0809 representing the isotopically labeled trimethyl nitrogen, and at least two other characteristic acyl-homocarnitine product ions (*m/z* 99.0441, 117.0546, 158.1176 (161.1276 with $^{13}C_3$)).

Supporting Information: Figure S18

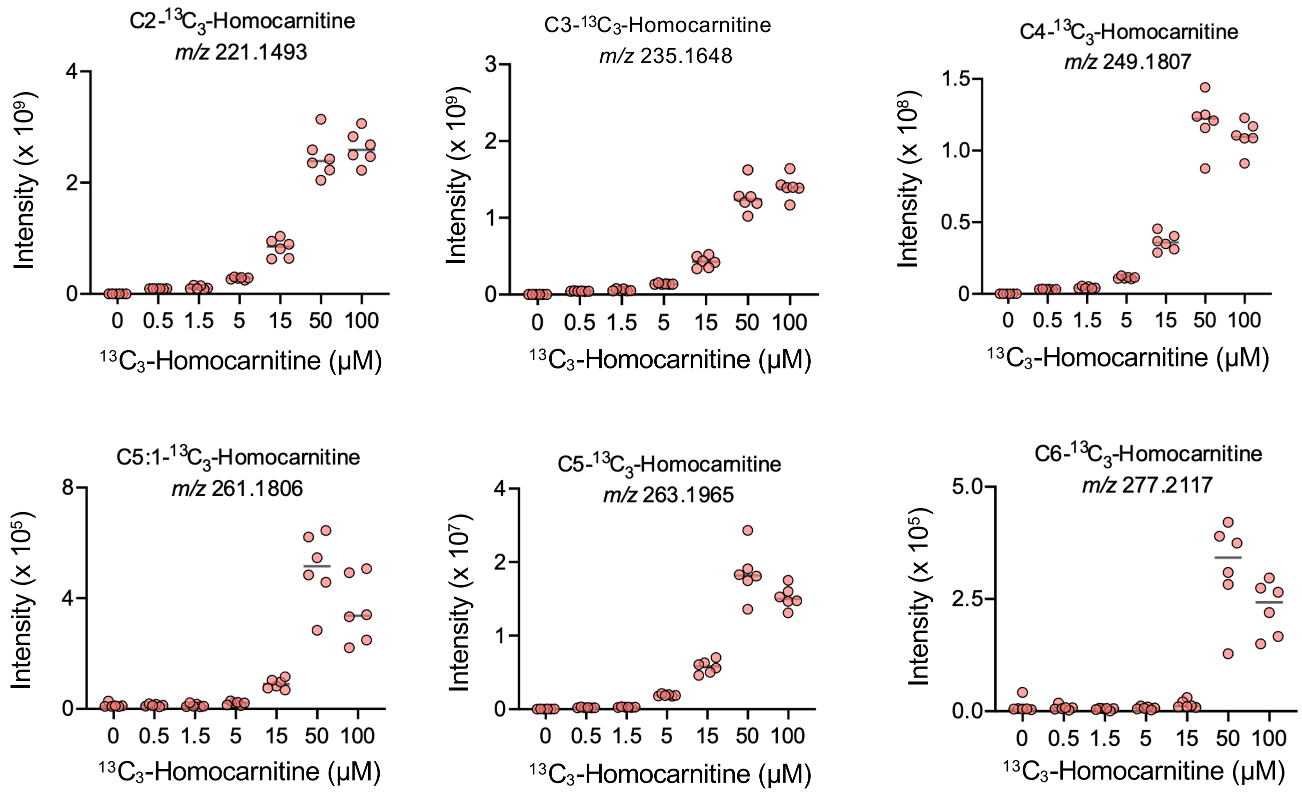

Figure S18. Dose-response $^{13}C_3$-homocarnitine tracer study in AC16 cells. Cells were treated with 0, 0.5, 1.5, 5, 15, 50 and 100 μM $^{13}C_3$-homocarnitine for 18 h (n = 6 for each). Signals matching a theoretical *m/z* for an acyl-$^{13}C_3$-homocarnitine. Annotation required an increase with treatment, a MS^2 product ion at *m/z* 63.0809 representing the isotopically labeled trimethyl nitrogen, and at least two other characteristic acyl-homocarnitine product ions (*m/z* 99.0441, 117.0546, 158.1176 (161.1276 with $^{13}C_3$)).

Supporting Information: Figure S19

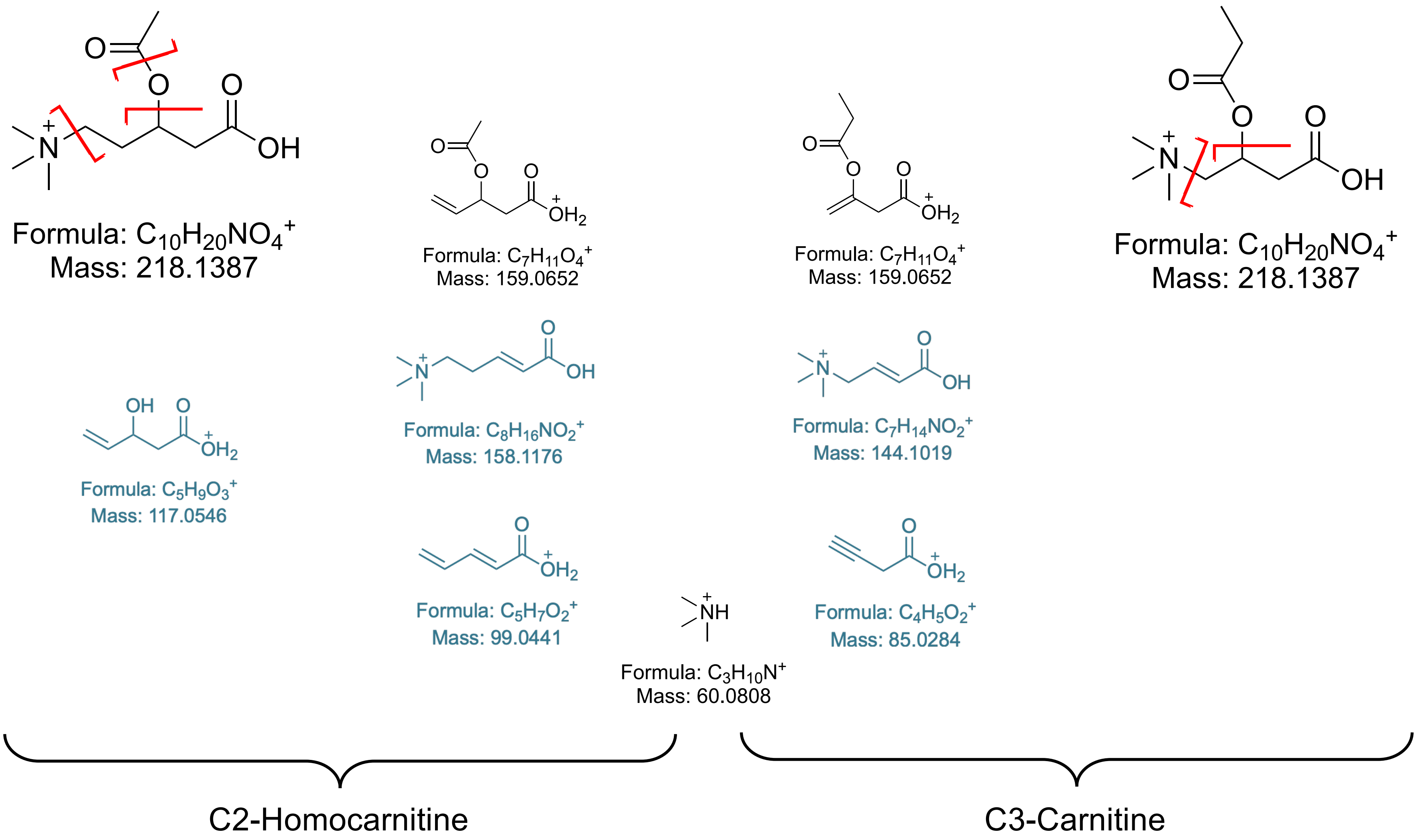

Figure S19. Proposed structures for the major MS² product ions of C2-homocarnitine and C3-carnitine, both *m/z* 218.1387. Sites of dissociation are indicated by the red lines. Analogous product ions for each are placed in the same row. Blue structures are differentiating product ions. Note: C3-carnitine has an analogous product ion at *m/z* 103.0390 for homocarnitine's *m/z* 117.0546, however it is typically very low abundant.

Supporting Information: Figure S20

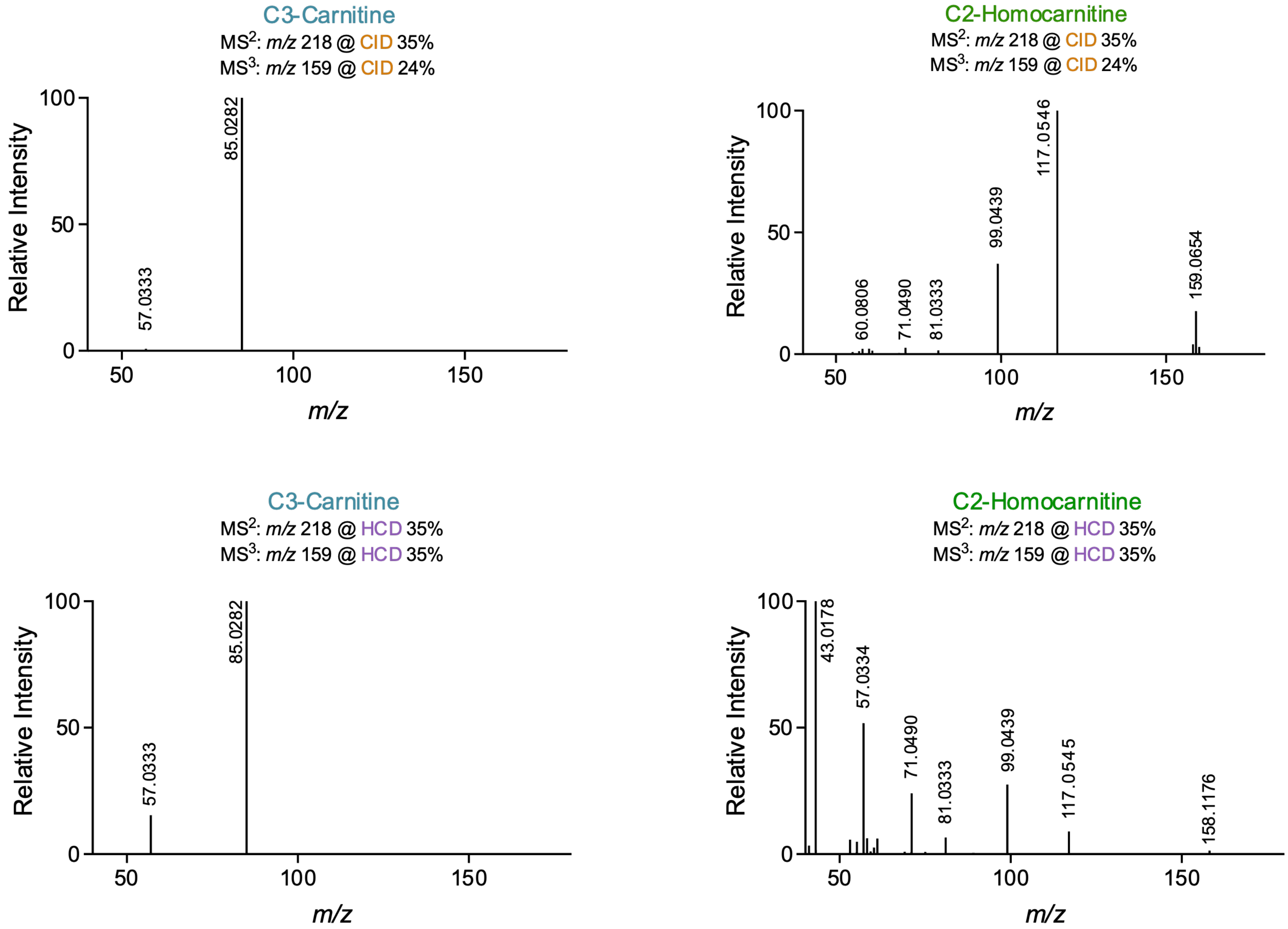

Figure S20. MS³ spectra of *m/z* 159.0654, a non-differentiating product ion of *m/z* 218.1387 (C2-homocarnitine or C3-carnitine). CID (top) and HCD (bottom).

Supporting Information: Figure S21

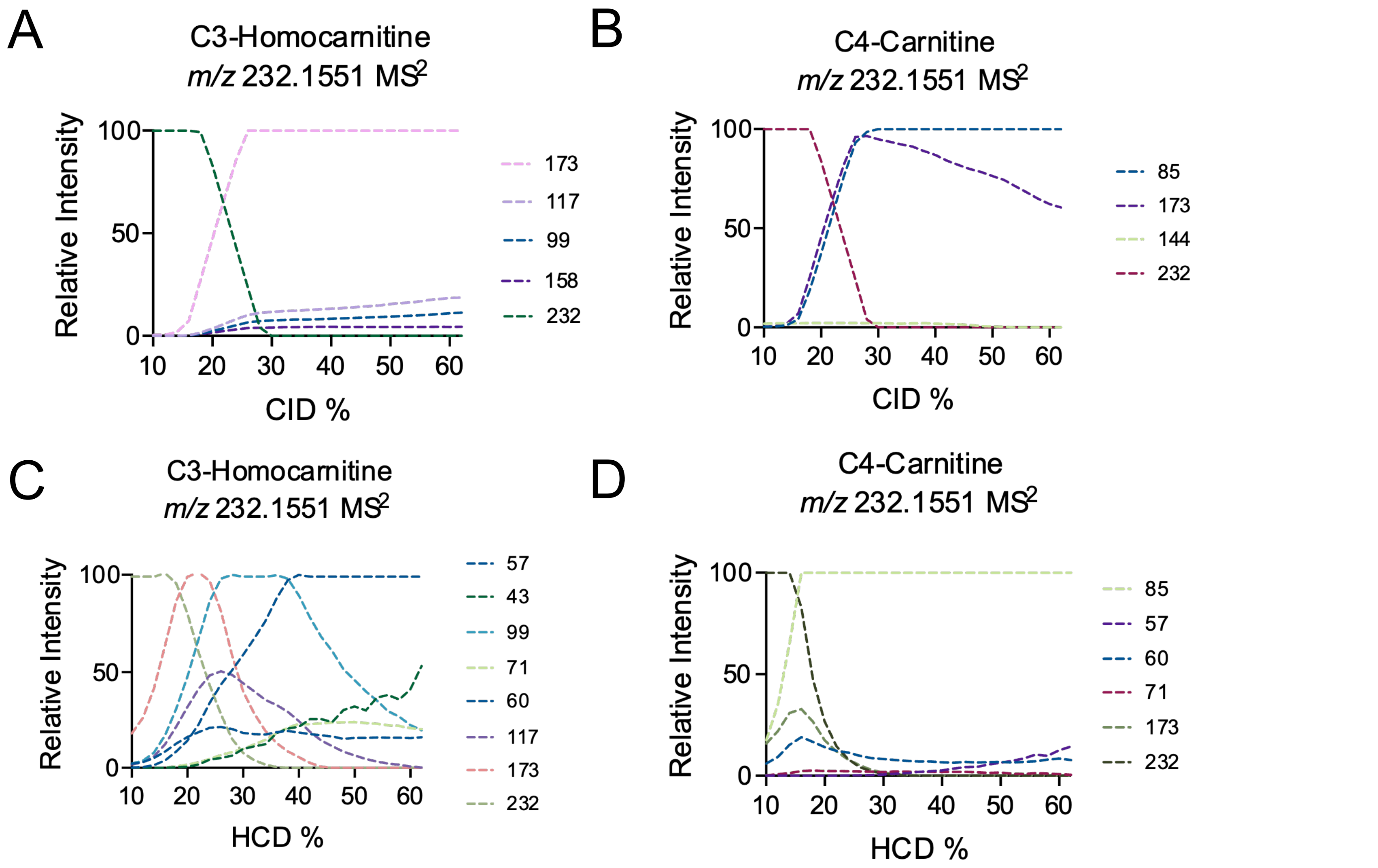

Figure S21. Energy-resolved mass spectrometry study of C3-homocarnitine and C4-carnitine isomers. Standards generated by carnitine acetyltransferase (CrAT) were analyzed with HILIC/ESI+ and normalized HCD or CID % was ramped in steps of 2 from 10-62. Relative intensities of product ions were documented. **A.** For C3-homocarnitine, increasing CID % revealed 5 major product ions. **B.** For C4-carnitine, increasing CID % revealed 4 major product ions. **C.** For C3-homocarnitine, increasing normalized HCD % revealed 8 major product ions. **D.** For C4-carnitine, increasing HCD % revealed 6 major product ions.

Supporting Information: Figure S22

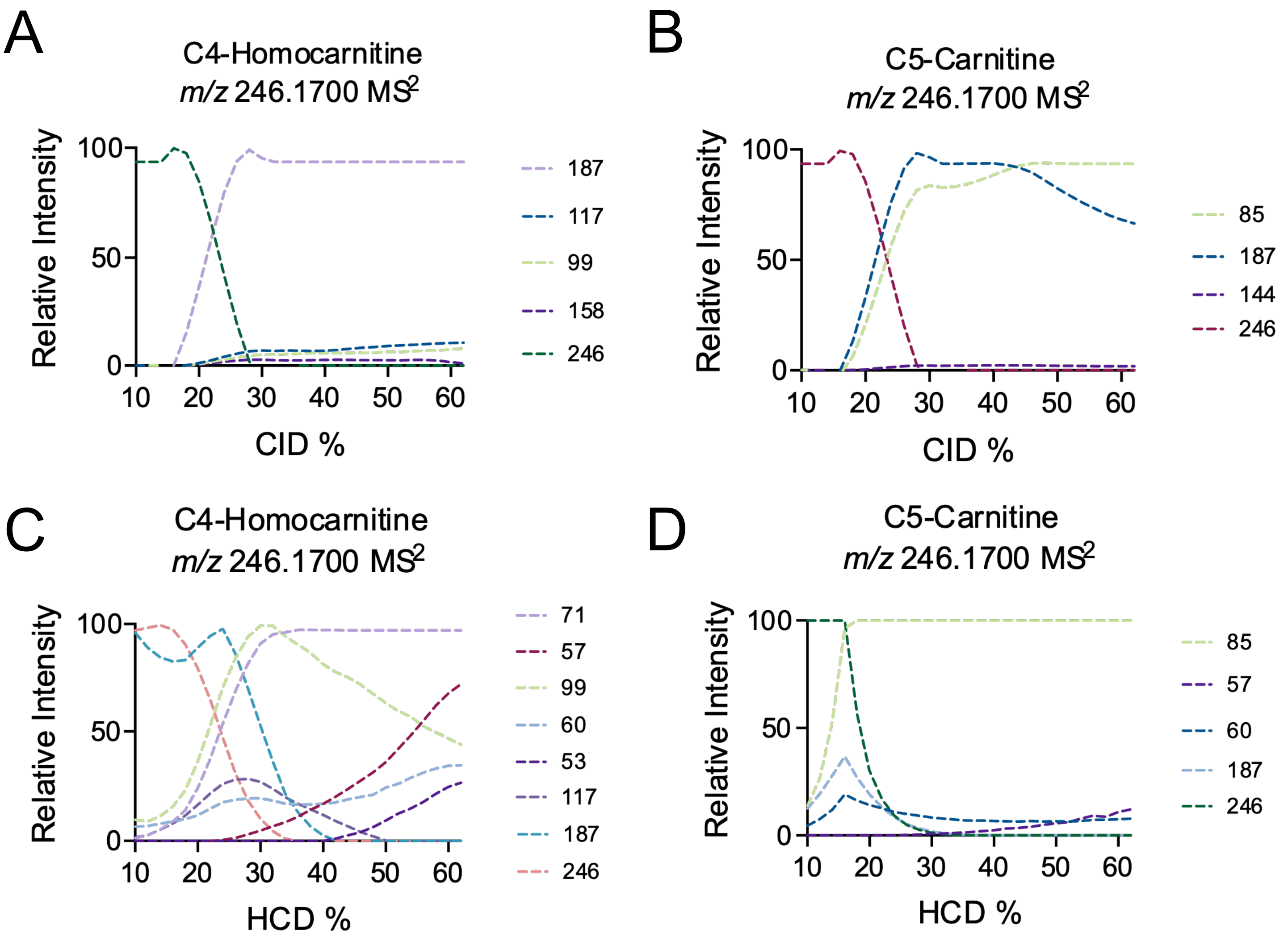

Figure S22. Energy-resolved mass spectrometry study of C4-homocarnitine and C5-carnitine isomers. Standards generated by carnitine acetyltransferase (CrAT) were analyzed with HILIC/ESI+ and normalized HCD or CID % was ramped in steps of 2 from 10-62. Relative intensities of product ions were documented. **A.** For C4-homocarnitine, increasing CID % revealed 5 major product ions. **B.** For C5-carnitine, increasing CID % revealed 4 major product ions. **C.** For C4-homocarnitine, increasing normalized HCD % revealed 8 major product ions. **D.** For C5-carnitine, increasing HCD % revealed 5 major product ions.

Supporting Information: Figure S23

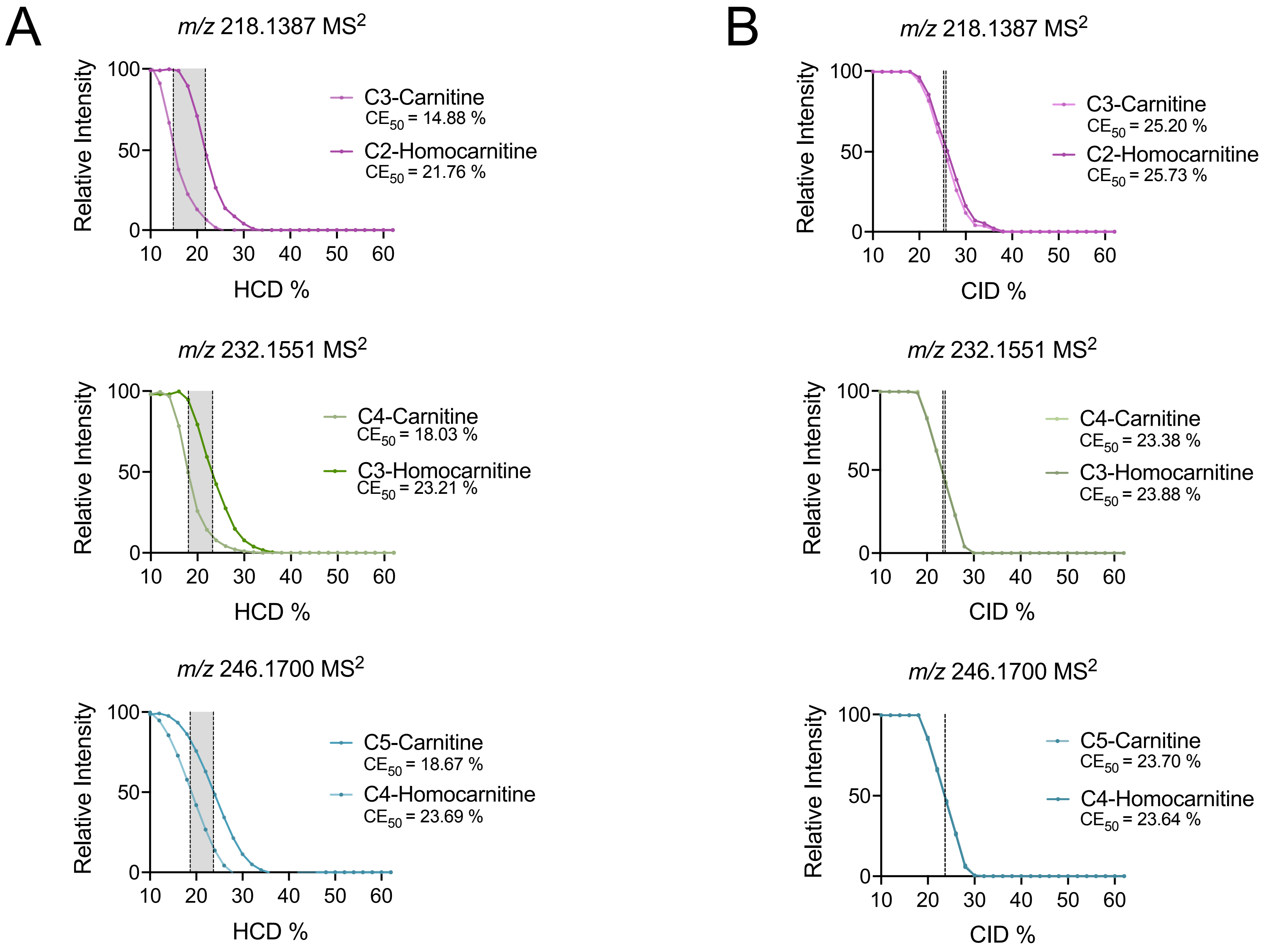

Figure S23. CE_{50} values for isomers under CID or HCD. A. Relative intensity of precursor ions plotted across increasing normalized HCD. **B.** Relative intensity of precursor ions plotted across increasing normalized CID. CE_{50} = normalized collision energy at which 50% of the precursor ion is degraded. Calculated by a nonlinear regression in GraphPad Prism (v.10.6.1). Shaded region highlights the difference of CE_{50} values between isomer pairs.

Supporting Information: Figure S24

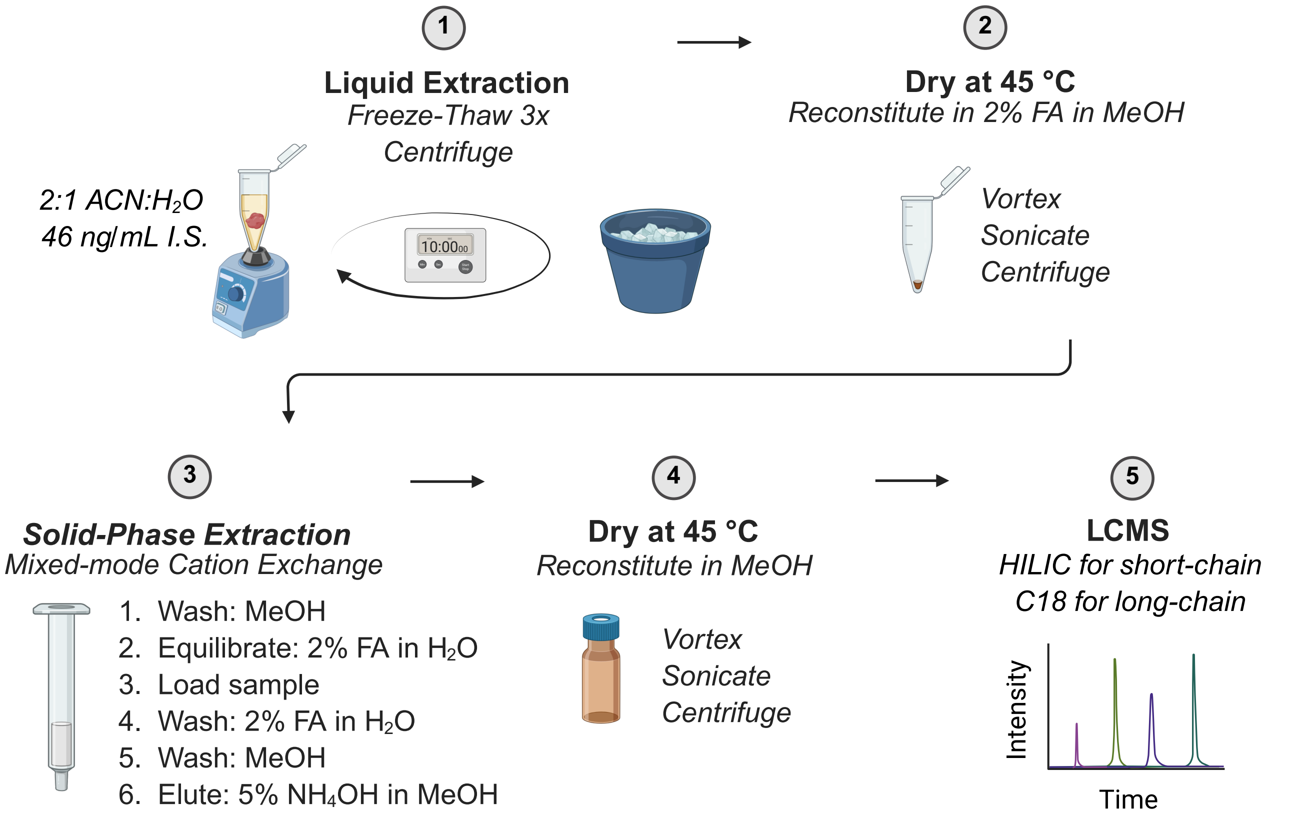

Figure S24. Sample preparation workflow for isolation of homocarnitine, carnitines, and betaines. Step 1. Liquid extraction of tissue with 3 freeze-thaw cycles on dry ice with acetonitrile (ACN): water (H2O) (2:1 ratio v/v) spiked with 46 ng/mL internal standard (I.S.). **Step 2.** Liquid extracts undergo centrifugation, the supernatant is dried, and the pellet is reconstituted in 2% formic acid (FA) in methanol (MeOH). Solubilized samples are vortexed, sonicated for 5 min, and undergo centrifugation. **Step 3.** Solid phase extraction tubes are washed and equilibrated with vacuum. Extracts are subjected to the mixed-mode cation cartridge, washed 2x, and eluted. **Step 4.** Extracts are dried, reconstituted in methanol, vortexed, sonicated for 5 min, and undergo centrifugation. Supernatants are transferred to autosampler vials. **Step 5.** HILIC method separates short-chain carnitine and homocarnitine isomers and resolves related betaines. C18 reverse-phase chromatography resolves long-chain acylated species. Created with Biorender.com.

Supporting Information: Figure S25

Group	Compound	Peak Area	*m/z*	Time (min)
Control	C5-Homocarnitine	4.49E+04	260.1856	4.09
δ-Valerobetaine	C5-Homocarnitine	1.76E+07	260.1856	4.09
Control	C6-Carnitine	1.81E+07	260.1856	3.33
δ-Valerobetaine	C6-Carnitine	9.14E+06	260.1856	3.33
Control	C4-Homocarnitine	1.67E+06	246.1702	4.32
δ-Valerobetaine	C4-Homocarnitine	8.16E+06	246.1702	4.32
Control	C5-Carnitine	9.89E+07	246.1702	3.60
δ-Valerobetaine	C5-Carnitine	9.70E+06	246.1702	3.60
Control	C3-Homocarnitine	1.48E+06	232.1545	4.64
δ-Valerobetaine	C3-Homocarnitine	1.29E+07	232.1545	4.64
Control	C4-Carnitine	1.43E+08	232.1545	3.81
δ-Valerobetaine	C4-Carnitine	6.50E+07	232.1545	3.81
Control	C2-Homocarnitine	1.51E+07	218.1388	4.96
δ-Valerobetaine	C2-Homocarnitine	1.48E+08	218.1388	4.96
Control	C3-Carnitine	2.16E+07	218.1388	4.13
δ-Valerobetaine	C3-Carnitine	1.84E+07	218.1388	4.13

Figure S25. Table of peak areas for acyl-homocarnitine and acyl-carnitine isomers in mouse heart.
